# Neurochemical and cognitive changes precede structural abnormalities in the TgF344-AD rat model

**DOI:** 10.1093/braincomms/fcac072

**Published:** 2022-03-25

**Authors:** Caitlin F. Fowler, Dana Goerzen, Gabriel A. Devenyi, Dan Madularu, M. Mallar Chakravarty, Jamie Near

**Affiliations:** 1 Department of Biological and Biomedical Engineering, McGill University, Duff Medical Building, Montreal, Canada H3A 2B4; 2 Centre d’Imagerie Cérébrale, Douglas Mental Health University Institute, Verdun, Canada H4H 1R3; 3 Department of Psychiatry, McGill University, Montreal, Canada H3A 1A1; 4 Centre for Translational NeuroImaging, Northeastern University, Boston, USA; 5 Physical Studies Research Platform, Sunnybrook Research Institute, Toronto, Canada M4N 3M5; 6 Department of Medical Biophysics, University of Toronto, Toronto, Canada M5G 1L7

**Keywords:** Alzheimer’s disease, MRI, magnetic resonance spectroscopy, neuroimaging biomarkers, TgF344-AD rat model

## Abstract

Alzheimer’s disease is a progressive neurodegenerative disorder with a decades-long pre-symptomatic phase, substantiating the need for prodromal biomarker development and early intervention. To deconstruct the processes underlying disease progression and identify potential biomarkers, we used neuroimaging techniques with high translational potential to human clinical studies in the TgF344-AD rat model which recapitulates the full spectrum of Alzheimer’s neuropathology (progressive amyloid deposition, tauopathy, frank neuronal loss, gliosis, and cognitive dysfunction). We employed longitudinal MRI and magnetic resonance spectroscopy in conjunction with behavioural testing to characterize multiple facets of disease pathology in male and female TgF344-AD rats (*n* = 26, 14M/12F) relative to wildtype littermates (*n* = 24, 12M/12F). Testing was performed at 4, 10, 16, and 18 months, covering much of the adult rat lifespan and multiple stages of disease progression. The TgF344-AD model demonstrated impaired spatial reference memory in the Barnes Maze by 4 months of age, followed by neurochemical abnormalities in the hippocampus by 10 months and major structural changes by 16 months. Specifically, TgF344-AD rats displayed increased total choline and lactate, and decreased total creatine, taurine, and N-acetylaspartate to myo-inositol ratio, dentate gyrus hypertrophy, and atrophy in the hippocampus, hypothalamus, and nucleus accumbens. Overall, these findings support the use of MRI and magnetic resonance spectroscopy for the development of non-invasive biomarkers of disease progression, clarify the timing of pathological feature presentation in this model, and contribute to the validation of the TgF344-AD rat as a highly relevant model for pre-clinical Alzheimer’s disease research.

## Teaser

Fowler *et al*. used brain imaging and cognitive testing in an Alzheimer’s disease rat model to define the chronological order in which changes to brain structure, tissue chemistry, and memory appear. Memory impairments were present prior to altered brain chemistry, both of which occurred before changes in brain volume.

## Graphical Abstract

**Graphical abstract:**
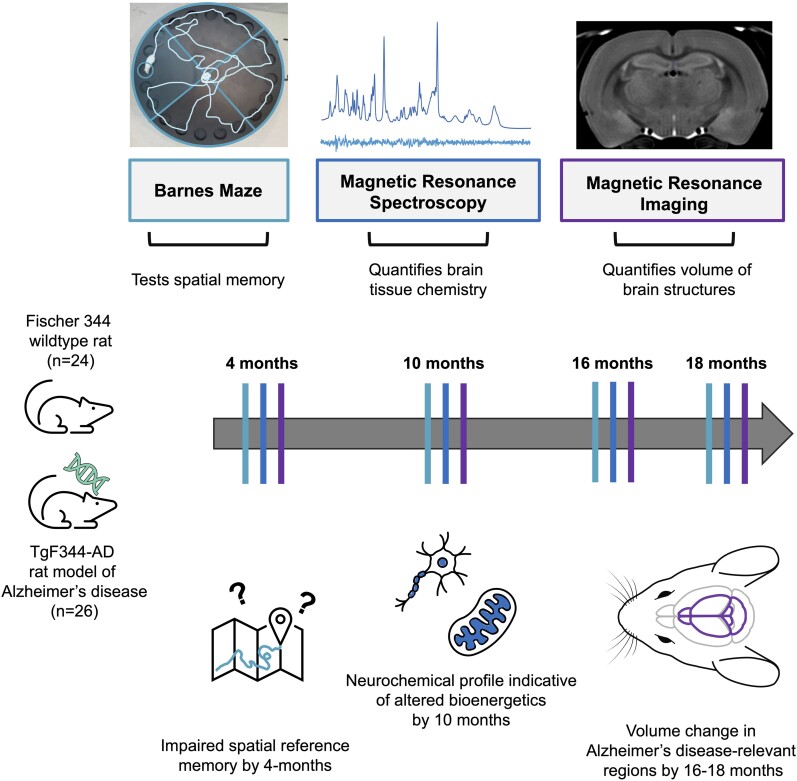

## Introduction

Alzheimer’s disease is a progressive neurodegenerative disorder that accounts for 60–80% of the 50 million dementia cases worldwide.^1^ Aspects of Alzheimer’s pathology can occur decades before clinical onset,^2–4^ substantiating the need for prodromal biomarker development and early intervention.

Early-stage disease characterization in transgenic animal models represents one promising avenue towards the development of new biomarkers and intervention approaches at a clinical level. The most common transgenic models are rodents expressing human genes harbouring mutations known to drive amyloid-β accumulation and cause familial or early-onset Alzheimer’s disease in humans, such as presenilin-1 (*PS1*), presenilin-2 (*PS2*), and amyloid precursor protein (*APP*).^5–7^ Amyloid-β-overproducing rodents are considered ‘gold standard’ models but most do not display robust tauopathy or neuronal loss—two major hallmarks of Alzheimer’s disease—unless additional human transgenes are expressed that are not associated with familial Alzheimer’s disease (typically microtubule-associated protein tau, *MAPT*^7,8^), as is the case with the widely used 3xTg mouse.^9^

To date, one of the only rodent models to recapitulate the full spectrum of Alzheimer’s disease neuropathology without insertion of a human tau transgene is the TgF344-AD rat which displays progressive amyloid-β deposition, tauopathy, gliosis, neuronal loss, and cognitive impairment, despite only expressing mutant human *APP* (APPswe, KM670/671NL) and *PS1* (ΔE9) genes.^10^ Not only does the TgF344-AD model represents a major advancement for Alzheimer’s disease research, but the numerous advantages of studying rats over mice—they are physiologically and genetically closer to humans, display a richer behavioural phenotype, and have larger brains^11^—make the TgF344-AD rat a particularly salient option for pre-clinical biomarker development.

Magnetic resonance (MR) techniques such as MRI and MR spectroscopy (MRS) enable non-invasive, longitudinal assays of brain structure and tissue chemistry at the pre-clinical and clinical level.^12–14^ MRI studies have identified reduced cortical thickness and atrophy of the medial temporal lobe as prominent features of Alzheimer’s disease in human subjects^13,15^ that may precede clinical diagnosis.^16,17^ Similar studies in transgenic models have replicated some of these findings.^18–22^ Proton MRS studies in rodent models of Alzheimer’s disease have identified altered metabolic profiles in the hippocampus, cingulate cortex, and frontal cortex, including reduced N-acetylaspartate (NAA) and glutamate (Glu), and increased myo-inositol (Ins), glutamine (Gln), and total choline (tCho).^23–26^ These changes parallel those observed in similarly affected brain regions in human Alzheimer’s patients such as the posterior cingulate and occipital cortices,^27,28^ while also informing on physiological processes involved in disease pathogenesis, including neuronal viability, cell membrane turnover, antioxidant capacity, neuroinflammation, energy metabolism, and neurotransmission.^29,30^ Despite the relevance of MRI and MRS in Alzheimer’s disease research, few studies have simultaneously examined the longitudinal progression of anatomical, neurochemical, and cognitive changes in either rodent models or humans. As such, a gap in knowledge exists regarding the relative timing of the appearance of these pathological features, limiting the understanding of disease stages and subsequent design of therapeutic approaches.

The aim of this study was to characterize the manifestation and time course of pathological change in neuroimaging biomarkers and cognition in the TgF344-AD rat *in vivo* to determine whether it recapitulates major features of human-AD and to contribute to better disease staging of this model. We employed MRI, MRS, and Barnes Maze testing at 4, 10, 16, and 18 months of age to distinguish longitudinal changes in neuroanatomy, neurochemistry, and cognitive function in male and female TgF344-AD rats relative to wildtype (WT) littermates.

## Materials and methods

### Animal care and study design

The TgF344-AD model [transgenic (Tg)] is a double Tg line created on a Fischer 344 background that expresses the ‘Swedish’ mutant human *APP* (*APP^swe^: APP KM670/671NL*) and deletion of exon 9 mutant of human *PS1* (*PS1ΔE9*). Male hemizygous TgF344-AD rats (Terrence Town Laboratory, University Southern California, CA, USA) and female homozygous Fischer 344/NHsd WT rats (Envigo, Madison, WI, USA) were bred in-house. Offsprings were a mixture of hemizygous Tg and homozygous WT rats. Tail snips were obtained from each rat to identify the presence of the *APP^swe^* and *PS1ΔE9 transgenes* (genotyping by Transnetyx, Memphis, TN, USA). Rats were weaned on post-natal Day 21 and housed in same-sex pairs on a 12 h light–dark cycle with *ad libitum* access to food (Envigo, Teklad Global 18% Protein Rodent Diet) and water. All animal procedures and experiments were performed in accordance with the guidelines of the local institutional Animal Care Committee.

In total, 24 WT rats (12M/12F) and 26 Tg rats (14M/12F) were studied longitudinally, with Barnes Maze testing and neuroimaging performed at 4, 10, 16, and 18 months of age. Behavioural testing was performed prior to neuroimaging to avoid confounds of anaesthesia on behaviour. Sample size calculations for comparison between genotypes while controlling for sex were performed using a population simulation-based power analysis tool^31^ and can be found in Supplementary Methods. We also present exploratory analyses of the intersecting effect of genotype and sex on all neuroimaging and behavioural markers. Group sizes at each time point are included in Supplementary Table 1.

### MRI data acquisition and regional volume estimation

MRI data were acquired using a 7 Tesla Bruker Biospec 70/30 scanner (Bruker, Billerica, MA, USA) with an 86 mm (diameter) volumetric birdcage coil for transmission and a four-channel surface array coil for signal reception (Bruker). The level of anaesthesia (1–4% isoflurane in oxygen gas) was adjusted to maintain a breathing rate between 50 and 75 breaths/min throughout the procedure and warm air (37°C) was blown into the bore of the scanner to maintain a constant body temperature (SA Instruments Inc., Monitoring System, Stony Brook, NY, USA).

High-resolution 3D anatomical MR images were acquired using Rapid Acquisition with Relaxation Enhancement using scan parameters identical to those described previously.^32,33^ Scan resolution was 114 µm isotropic and images were T1-weighted. All pre-processing methodology is described in detail elsewhere^32^ and in Supplementary Methods. After pre-processing, images were examined for motion artefacts, Gibbs ringing artefacts, and other image anomalies, following which 15 of a total 179 scans were excluded from further analysis. Seven rats [four Tg (1M/3F) and three WT (2M/1F)] were excluded at 4 months, one at 10 months [1 Tg (M)], and five at 16 months [three Tg (1M/2F), two WT (1M/1F)]. The remaining 164 scans were co-registered using the two-level deformation-based morphometry pipeline in Pydpiper, as described by Friedel *et al*. ^34^ and in Supplementary Methods. This process creates deformation fields for each subject at each time point, reflecting the amount of expansion or compression required to deform each individual anatomical image to the subject average.^35^ Deformation fields are then resampled into the common study space allowing comparison between subjects. The Fischer 344 rat atlas was used to estimate the volume of 120 regions.^36^

### Proton MRS data acquisition and quantification

Immediately following MRI data acquisition, MRS data acquisition was performed using the same methodology as described previously.^37^ Automated localized shimming was performed using the FASTMAP method ^38^ (ParaVision 5.1, Bruker). Proton MRS scans were acquired from a 2.5 mm × 3.5 mm × 3.5 mm voxel in the dorsal hippocampus using a Point RESsolved Spectroscopy sequence (acquisition time = 13m0s0ms, TR = 3000 ms, TE = 11.12 ms, 2048 acquisition data points, spectral width = 4006 Hz) in combination with outer volume suppression. In total, 256 averages were acquired with VAPOR water suppression^39^ and 8 averages were acquired without water suppression for eddy current correction and as a reference for absolute metabolite quantification.

Spectral pre-processing was performed in the FID-A toolbox (github.com/CIC-methods/FID-A, version 1.0^40^) in MATLAB (R2012a, The MathWorks Inc., Natick, MA, USA), and consisted of removal of motion-corrupted scans and spectral registration to correct frequency and phase drift errors. Processed spectra were analysed using LCModel (version 6.3, Stephen Provencher Inc., Oakville, Ontario, Canada)^41^, with a neurochemical basis set consisting of 18 simulated metabolite resonances and 9 macromolecule basis functions. Methods detailing the acquisition of macromolecule spectra for parameterization and inclusion into the quantification basis set are described elsewhere.^37^

Absolute quantification was performed using the unsuppressed water signal as a reference. A correction was applied to account for T1 and T2 relaxation constants of water and measured neurochemicals, and an assumed NMR-visible water concentration of 4300 mM given that our voxel contained mostly grey matter (GM).^42^ For details on the correction formula, see the supplementary material in our previous publication.^37^ Neurochemical concentrations are reported in mmol/L (mM). Details regarding the basis set and quality control methods are included in Supplementary Methods.

### Behavioural phenotyping via the Barnes Maze test

We assessed hippocampus-dependent spatial reference memory using a shortened variation^43^ of the popular Barnes Maze protocol.^44^ Detailed methodology is described in Supplementary Methods. Briefly, a circular maze with 20 holes was used, and rats were trained to locate a single escape hole that led to a box underneath. Rats were given three 3-min trials on Day 1 and two 3-min trials on Day 2, for a total of five training trials. A probe trial was used to test long-term spatial reference memory. The probe trial was conducted 48 h after the last training trial and involved blocking the escape hole so that no escape was possible. All sessions were recorded using a Logitech QuickCam Pro 9000. The following metrics were measured during the probe trial using EthoVision XT Software (Noldus Information Technology, Wageningen, The Netherlands): % time in target quadrant, % time in target holes, success or failure to locate the escape hole, average speed (cm/s), and number of holes searched.

### Statistical analysis

Statistical analyses and visualizations were performed in R (version 3.6.3^45^). Brain volume and metabolite concentration data were modelled using linear mixed-effects models as they appropriately model the covariance structure resulting from repeated measurements in the same subjects and handle data with missing values.^46^ Brain volumes were predicted by a quadratic age-by-genotype interaction (Model 1), and metabolite concentrations were predicted by a linear age-by-genotype interaction (Model 2), with sex covaried and a random intercept for each subject. Genotype effects were evaluated as a group effect of Tg rats relative to WT rats at each time point using four age-centred models, with age centred at the average cohort age (129.6, 310.7, 494.3, and 572.9 days). All continuous variables were *z*-scored. For MRS data, the fixed effect of water linewidth was included to control for the effect of linewidth on metabolite concentration estimates.^47^ A weighting factor of the inverse absolute Cramer-Rao Lower Bound (CRLB) for each metabolite accounted for differences in fitting reliability between samples. We also examined a three-way interaction of age-by-genotype-by-sex with the same covariates as mentioned above for brain volumes (Model 3) and metabolite concentrations (Model 4).

For all linear models, the false discovery rate (FDR) method^48^ was used to control the family-wise Type I error at a level of 5% for each predictor of interest. Details on attached base packages in R, Akaike information criterion comparisons, and Linear Models 1 through 4 are included in Supplementary Methods.

Barnes Maze data were analysed cross-sectionally. % success, number of holes searched, and speed were analysed using a linear model with genotype as a fixed effect and sex covaried, or genotype and sex interacting (secondary analysis). % time in the target quadrant and % time in target holes were assessed using a one-sample *t*-test or a Wilcoxon signed rank test (if test residuals were non-normal) against a mean of 25% (chance amount of time) within WT and Tg rats, as well as for genotypes split by sex (WT males, WT females, Tg males, and Tg females). Bonferroni correction was applied at each time point for the primary and secondary analyses separately, whereby the *P*-value threshold was set at 0.05/7 tests (*P* < 0.00714) or 0.05/11 tests (*P* < 0.00455), respectively.

### Data availability

Data are reported within the text, figures, and supplementary material. Raw data are published to the publicly available repository, Zenodo, at 10.5281/zenodo.6338797.

## Results

### TgF344-AD rats display altered local brain volume, primarily in GM structures

**Figure 1:**
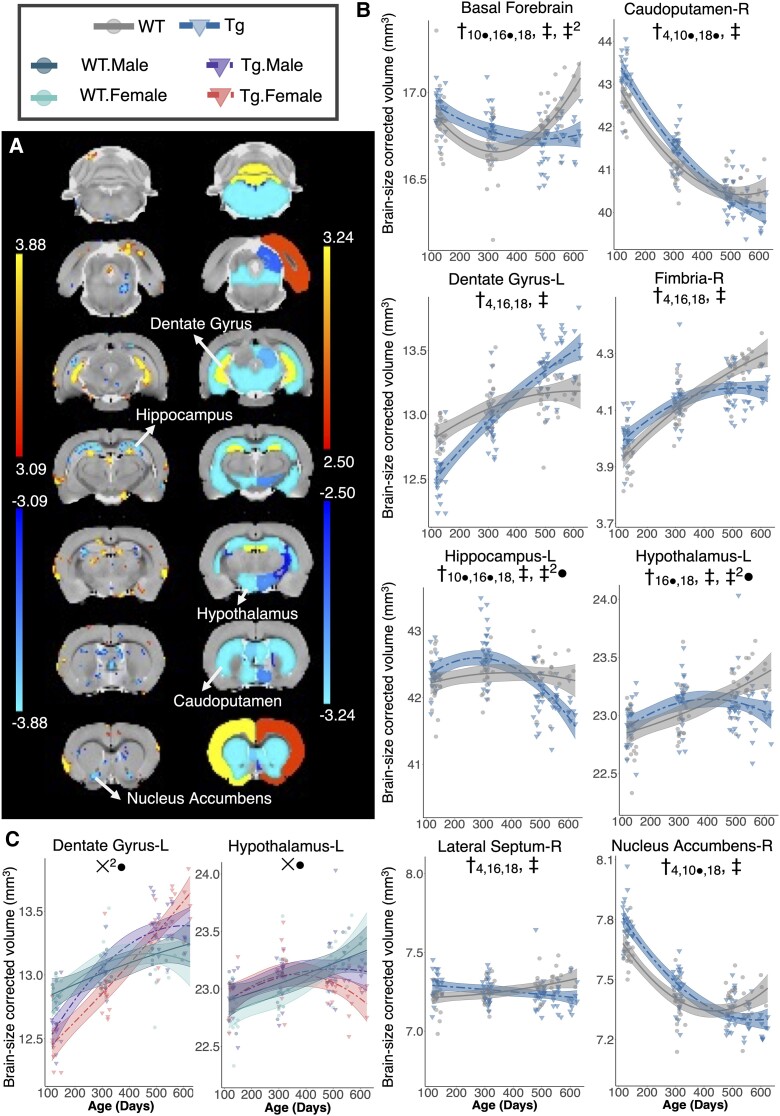
**Genotype-dependent differences in local brain volume with age**. (**A**) Voxel-wise (left) and regional (right) statistical maps for the linear age-by-genotype interaction term are shown. The plot range for each set of t-values displays an interaction significant between 5 and 1% FDR, with *t*-values corresponding to FDR < 1% displayed at the 1% value. Positive *t*-values (warm colours) represent increasing volume in TgF344-AD rats over time relative to WT littermates (positive age-by-genotype interaction), whereas negative numbers (cool colours) represent decreasing volume in TgF344-AD rats (negative age-by-genotype interaction). Select regional volume trajectories in WT and TgF344-AD (Tg) rats are shown in (**B**). The mixed-effects model used to fit the data is represented by a line of best fit and 95% prediction interval (shaded). Significance symbols are shown for the linear age-by-genotype interaction term (‡) and quadratic age-by-genotype interaction term (‡^2^). The main effect of genotype as determined by the four age-centred models is shown by (†), with the subscript denoting at which age the main effect was significant. (**C**) A three-way interaction between age, genotype, and sex was also explored, with a 5% FDR correction applied. Significance symbols for the linear and second order age-by-genotype-by-sex interaction terms are denoted by *X* and *X*^2^, respectively. ● denotes an effect significant at the *P*-value level but not after FDR correction.

Volume changes for the age-by-genotype interaction term of Model 1 are illustrated as *t*-statistic maps in Fig. 1A for voxel-wise *(left)* and regional *(right)* analyses. Significant effects were generally consistent between the two methods and the majority were bilateral. Brain structures demonstrating significant age-by-genotype interactions are summarized in Table 1. Of the significant interactions, 19 occurred in GM regions, 7 in white matter (WM) regions, and one in the ventricular system. Most interactions (16 of 19 for GM, 5 of 7 for WM) were negative, indicating decreased volume with age in Tgs relative to WTs. The strongest interactions were atrophy in the basal forebrain, caudoputamen, fimbria, hippocampus, and nucleus accumbens, unilateral atrophy in the right fornix, and hypertrophy in the dentate gyrus. Weaker effects were present as increases in cerebellar WM and aqueduct volume, and decreased ventral pallidum, lateral septum, and hypothalamus volume. The basal forebrain was the only structure to demonstrate a significant quadratic interaction, indicating different curvilinearity in the volume trajectory of Tg rats relative to WTs. Whole-brain GM, WM, and CSF volumes were also quantified but did not differ by genotype.

**Table 1:** Linear mixed-effects model summary for brain structures demonstrating significant effects

	Age:genotypeTg		GenotypeTg (4 months)		GenotypeTg (10 months)		GenotypeTg (16 months)		GenotypeTg (18 months)	
	Beta ± SE	*P*-value	Beta ± SE	*P*-value	Beta ± SE	*P*-value	Beta ± SE	*P*-value	Beta ± SE	*P*-value
Aqueduct	6.0 ± 1.8	**9.9E-04**	−0.8 ± 0.3	**4.6E-03**	−0.4 ± 0.2	9.4E-02	0.2 ± 0.2	3.5E-01	0.5 ± 0.3	9.1E-02
Basal forebrain	−8.3 ± 1.7	**2.1E-06**	0.4 ± 0.3	9.4E-02	0.8 ± 0.2	8.9E-04	−0.4 ± 0.2	2.3E-02	−1.4 ± 0.3	**7.1E-07**
Bed nucleus of the stria Terminalis-L	−5.3 ± 1.6	**1.0E-03**	0.67 ± 0.2	7.6E-03	0.3 ± 0.2	1.6E-01	−0.2 ± 0.2	2.3E-01	−0.5 ± 0.3	6.7E-02
Caudoputamen-R	−3.8 ± 0.7	**4.0E-07**	0.5 ± 0.1	**1.1E-04**	0.3 ± 0.1	5.4E-03	−0.08 ± 0.1	4.2E-01	−0.3 ± 0.1	1.5E-02
Cerebellar WM	6.8 ± 1.5	**1.4E-05**	−0.9 ± 0.2	**3.0E-04**	0.03 ± 0.2	8.9E-01	0.5 ± 0.2	**2.5E-03**	0.5 ± 0.2	2.3E-02
Dentate gyrus-R	9.7 ± 1.1	**4.7E-15**	−1.2 ± 0.2	**1.5E-10**	−0.2 ± 0.2	1.5E-01	0.6 ± 0.1	**2.5E-06**	0.9 ± 0.2	**2.7E-06**
Fimbria-R	−6.6 ± 1.1	**8.7E-08**	0.5 ± 0.2	**4.0E-03**	0.2 ± 0.2	2.4E-01	−0.5 ± 0.1	**1.7E-04**	−0.9 ± 0.2	**3.1E-06**
Fornix-R	−10.2 ± 1.7	**1.3E-04**	0.9 ± 0.3	**4.9E-04**	0.1 ± 0.2	5.6E-01	−0.8 ± 0.2	**1.9E-05**	−1.2 ± 0.3	**1.1E-05**
Frontal cortex-L	3.4 ± 0.8	**2.6E-05**	−0.3 ± 0.1	2.5E-02	0.06 ± 0.1	6.3E-01	0.3 ± 0.1	**3.4E-03**	0.4 ± 0.1	**4.4E-03**
Hippocampus-R	−7.5 ± 1.7	**2.7E-05**	0.3 ± 0.3	2.7E-01	0.3 ± 0.2	2.3E-01	−0.6 ± 0.2	**5.1E-04**	−1.3 ± 0.3	**2.9E-06**
Hypothalamus-L	−6.3 ± 1.7	**2.8E-04**	0.2 ± 0.3	3.6E-01	0.4 ± 0.2	5.4E-02	−0.4 ± 0.2	1.8E-02	−1.1 ± 0.3	**4.0E-05**
Intrabulbar part of the anterior commissure-R	−6.7 ± 1.7	**1.3E-04**	0.8 ± 0.3	**2.6E-03**	0.06 ± 0.2	7.9E-01	−0.5 ± 0.2	1.2E-02	−0.6 ± 0.3	2.6E-02
Lateral olfactory tract-L	5.1 ± 1.7	**3.3E-03**	−1.0 ± 0.3	**3.6E-04**	0.07 ± 0.2	7.5E-01	0.3 ± 0.2	1.4E-01	0.08 ± 0.3	7.6E-01
Lateral septum-R	−8.9 ± 1.9	**4.1E-06**	0.8 ± 0.3	**4.8E-03**	0.3 ± 0.3	2.9E-01	−0.6 ± 0.2	**2.6E-03**	−1.1 ± 0.3	**3.7E-04**
Nucleus accumbens-L	−5.5 ± 1.1	**2.1E-06**	0.6 ± 0.2	**1.4E-03**	0.6 ± 0.2	**2.4E-04**	−0.1 ± 0.1	3.1E-01	−0.6 ± 0.2	**5.1E-04**
Olfactory nuclei-R	−7.2 ± 1.7	**5.7E-05**	0.8 ± 0.3	**1.9E-03**	0.5 ± 0.2	2.3E-02	−0.2 ± 0.2	2.2E-01	−0.7 ± 0.3	1.3E-02
Stria terminalis-R	−5.9 ± 1.4	**4.3E-05**	0.5 ± 0.2	1.5E-02	0.1 ± 0.2	4.6E-01	−0.4 ± 0.1	**4.5E-03**	−0.7 ± 0.2	**1.4E-03**
Superior colliculus-R	−5.6 ± 1.9	**3.2E-03**	1.1 ± 0.3	**1.7E-04**	−0.2 ± 0.3	4.0E-01	−0.3 ± 0.2	8.5E-02	−0.02 ± 0.3	9.3E-01
Ventral pallidum-R	−6.1 ± 1.6	**2.1E-04**	0.55 ± 0.25	2.62E-02	0.3 ± 0.2	1.3E-01	−0.3 ± 0.2	5.3E-02	−0.7 ± 0.3	**3.8E-03**

Linear age-by-genotype interactions and main effects of genotype are shown under age:genotypeTg and genotypeTg (age) columns, respectively. For concision, if effects were seen bilaterally, only one side (left, L; right, R) is displayed. Betas are standardized and SE denotes the standard error of the beta estimate. Unadjusted *P*-values are shown, with bold font denoting *P*-values that were still significant after applying an FDR correction at 5%. WM, white matter.

Four age-centred models examining the main effect of genotype at each time point were used to provide group difference snapshots of the timeline along which structural changes occur in the TgF344-AD model. As shown in Table 1, volume differences between WTs and Tgs were present at 4 months for the aqueduct, caudoputamen, dentate gyrus, nucleus accumbens, and fimbria. Hippocampal atrophy was not significant until 16 and 18 months of age and was preceded by a period of marginally increased volume relative to WTs. Volume trajectories for selected structures are shown in Fig. 1B.

Figure 1C shows trajectories for two structures split by sex and depicts results from the analysis using a three-way interaction between quadratic age, genotype, and sex (Model 2) to predict regional volume. The linear age-by-genotype-by-sex interaction term for the left hypothalamus was negative and the left dentate gyrus demonstrated a positive quadratic age-by-genotype-by-sex effect. However, neither these effects nor any other structures evaluated with Model 2 survived FDR correction.

A summary of linear model results for brain regions analysed using Models 1 and 2 is shown in Supplementary Table 2. Brain volumes in mm^3^ at each time point, both collapsed across and split by sex, are summarized in Supplementary Table 3. Trajectories of brain structures showing a significant age-by-genotype interaction via Model 1 can be found in Supplementary Figs 1 and 2. Those demonstrating significant three-way interactions via Model 2 (prior to FDR correction) are shown in Supplementary Figs 3 and 4.

**Figure 2:**
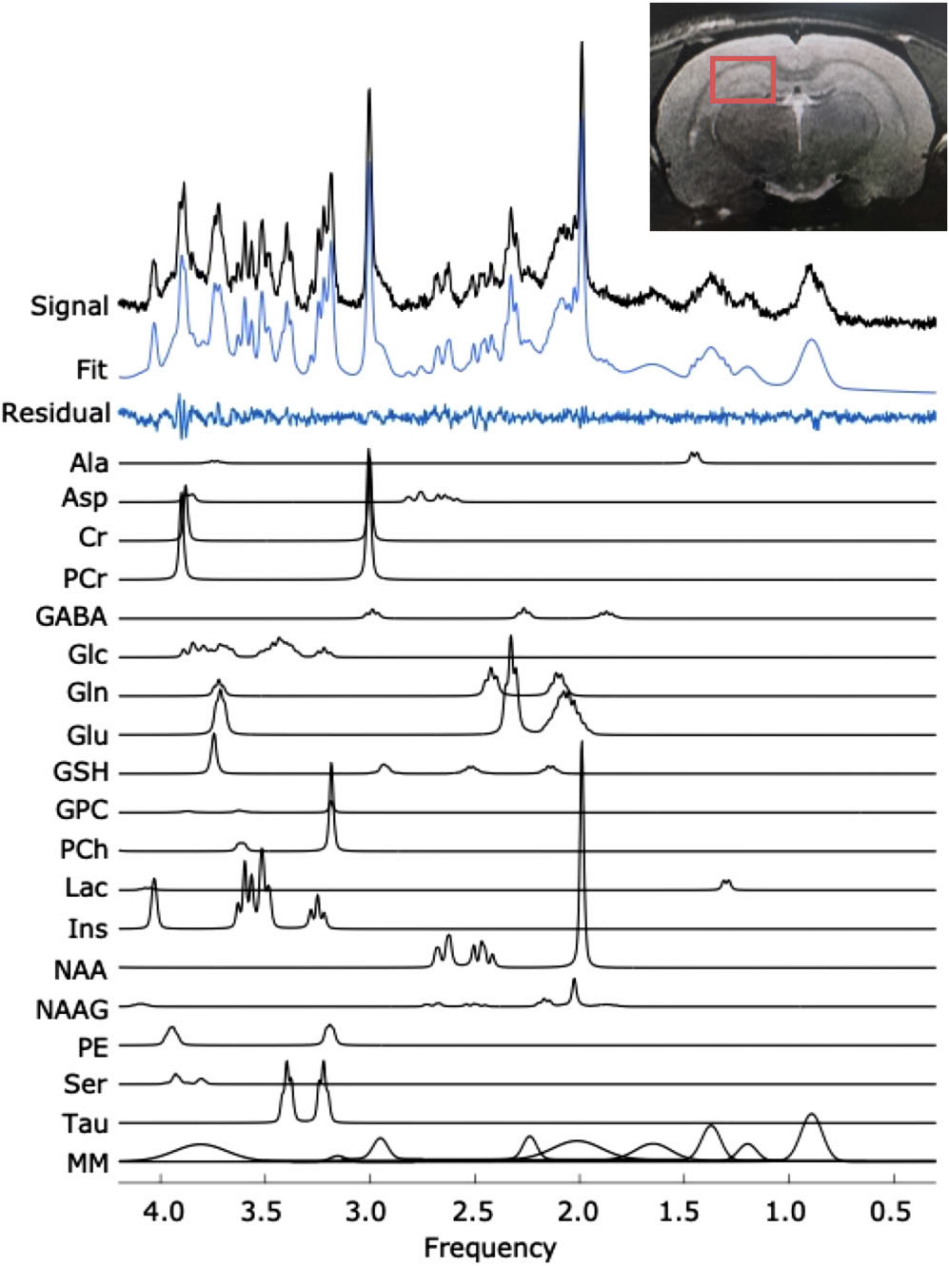
**A representative MRS spectrum showing data quality, fit, and basis functions.** A representative MRS spectrum obtained from a female TgF344-AD rat at 4 months, with individual metabolite and macromolecule fit components shown below. The vertical scale is in arbitrary units. The inset image shows the positioning of the voxel around the hippocampus.

### The TgF344-AD model recapitulates neurochemical features of Alzheimer’s disease

A total of 27 hippocampal neurochemicals were quantified longitudinally in Tg rats relative to WT littermates. The neurochemical profile contained nine macromolecule resonances which have yet to be quantified in this model. High-quality spectra were consistently obtained, as shown by the representative spectrum obtained from a 10-month-old WT female (Fig. 2), and by the low % CRLB values shown in Supplementary Table 4. The average signal-to-noise ratio of the NAA peak at 2.02 ppm was 61.77 [±13.96 (± standard deviation), range: 24.51–107.35], and the average linewidth of water was 9.21 Hz (±0.73, range: 7.74–12.79; measured as the full width at half max of the unsuppressed water peak in the reference scan).

**Figure 3:**
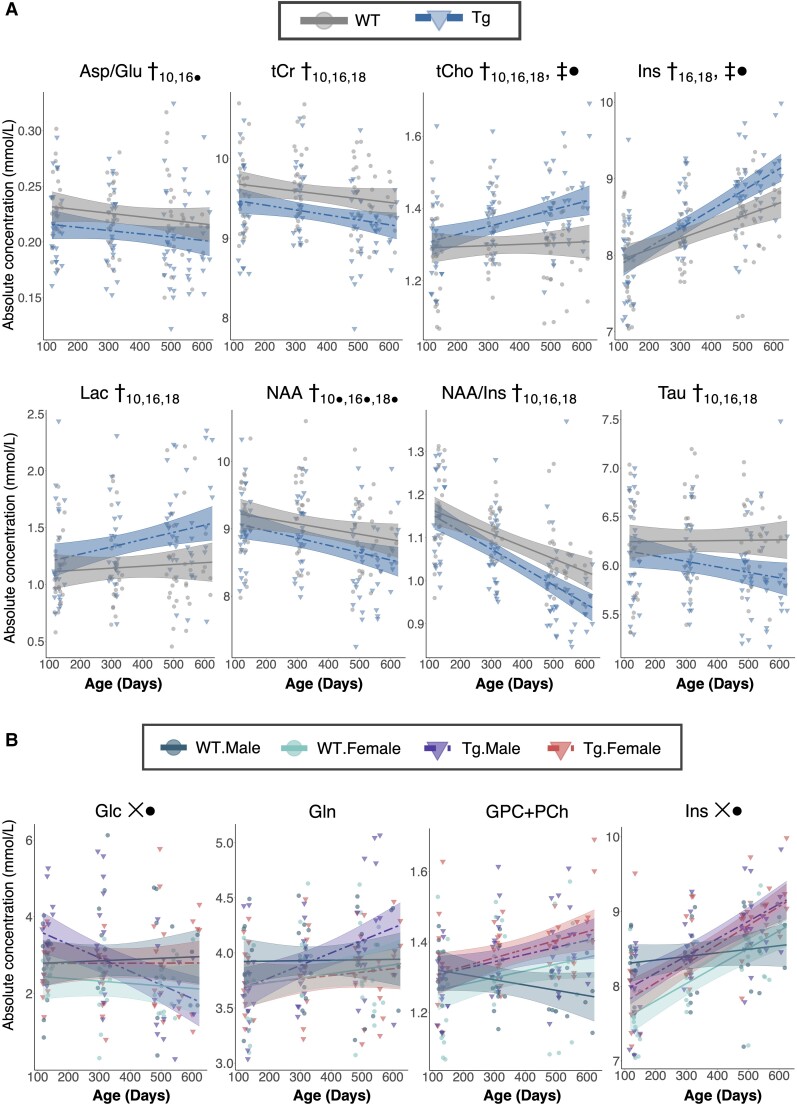
**Trajectory of neurochemical changes with age in TgF344-AD and WT rats**. (**A**) Select neurochemical concentration trajectories in WT and TgF344-AD (Tg) rats are shown. The mixed-effects model used to fit the data is represented by a line of best fit and 95% prediction interval (shaded). Significance symbols are shown for the linear age-by-genotype interaction term (‡), and the main effect of genotype (†) at each time point as determined by age-centred models. The subscript denotes the age at which the genotype effect was significant. (**B**) A three-way interaction between age, genotype, and sex was also explored, with a 5% false discovery rate correction applied. A significant three-way interaction term is denoted by X. ● denotes an effect significant at the *P*-value level but not after FDR correction.

The primary analysis explored the interaction between linear age and genotype while controlling for sex (Model 3). As shown in Table 2 and Fig. 3A, tCho and Ins demonstrated significant positive age-by-genotype interactions, whereby metabolite concentration increased more steeply with age in Tg rats than in WTs, but did not survive FDR correction. Upon examining the main effect of genotype at each time point using age-centred models, several metabolites differed between WT and Tg rats, with the earliest differences detected at 10 months (Table 2). Total creatine (tCr), taurine, and the ratio of NAA to Ins (NAA/Ins) were decreased in Tg rats at 10 months of age and remained lower at 16 and 18 months, whereas the ratio of aspartate (Asp) to Glu (Asp/Glu) was significantly lower at 10 months only. NAA was significantly lower at 10, 16, and 18 months but not after FDR correction. Higher concentrations of lactate (Lac) and tCho were evident at 10, 16, and 18 months, while Ins was significantly higher in Tg rats only at 16 and 18 months of age. None of the macromolecule peaks differed between Tg and WT rats.

**Table 2:** Linear mixed-effects model summary for all quantified neurochemicals

	Age:genotypeTg		GenotypeTg (4 months)		GenotypeTg (10 months)		GenotypeTg (16 months)		GenotypeTg (18 months)	
	Beta ± SE	*P*-value	Beta ± SE	*P*-value	Beta ± SE	*P*-value	Beta ± SE	*P*-value	Beta ± SE	*P*-value
Alanine	−0.03 ± 0.2	8.5E-01	0.1 ± 0.2	6.6E-01	0.08 ± 0.2	6.2E-01	0.05 ± 0.2	8.1E-01	0.03 ± 0.2	8.9E-01
Aspartate	0.03 ± 0.2	8.6E-01	−0.4 ± 0.3	1.5E-01	−0.3 ± 0.2	4.4E-02	−0.3 ± 0.2	1.3E-01	−0.3 ± 0.2	2.4E-01
Cr + PCr	−0.06 ± 0.13	6.5E-01	−0.4 ± 0.2	6.9E-02	−0.5 ± 0.1	**1.7E-03**	−0.5 ± 0.1	**2.6E-03**	−0.6 ± 0.1	**9.6E-03**
Glucose	−0.2 ± 0.1	1.4E-01	0.5 ± 0.3	5.3E-02	0.3 ± 0.2	1.5E-01	0.04 ± 0.2	8.7E-01	−0.06 ± 0.2	8.2E-01
Glutamine	0.2 ± 0.2	2.0E-01	−0.3 ± 0.2	2.2E-01	−0.1 ± 0.2	5.1E-01	0.1 ± 0.2	5.9E-01	0.2 ± 0.2	4.3E-01
Glutamate	−0.01 ± 0.1	9.7E-01	0.2 ± 0.2	5.3E-01	0.2 ± 0.1	3.3E-01	0.1 ± 0.1	4.5E-01	0.1 ± 0.1	5.5E-01
Glu + Gln	0.1 ± 0.2	4.4E-01	−0.05 ± 0.3	8.4E-01	0.07 ± 0.2	6.4E-01	0.2 ± 0.2	3.0E-01	0.3 ± 0.2	3.0E-01
GPC + PCh	0.3 ± 0.1	4.2E-02	0.2 ± 0.2	4.4E-01	0.5 ± 0.1	**1.6E-03**	0.8 ± 0.1	**3.3E-05**	0.9 ± 0.1	**7.9E-05**
GSH	0.1 ± 0.1	4.4E-01	−0.2 ± 0.2	4.8E-01	−0.05 ± 0.1	7.3E-01	0.06 ± 0.1	7.1E-01	0.1 ± 0.1	6.1E-01
Myo-Inositol	0.3 ± 0.1	2.2E-02	−0.04 ± 0.2	8.5E-01	0.2 ± 0.1	8.3E-02	0.5 ± 0.1	**2.0E-03**	0.6 ± 0.1	**1.6E-03**
Lactate	0.2 ± 0.1	1.3E-01	0.2 ± 0.2	3.3E-01	0.5 ± 0.2	**6.1E-03**	0.7 ± 0.2	**6.6E-04**	0.8 ± 0.2	**1.3E-03**
NAA	−0.1 ± 0.1	4.4E-01	−0.2 ± 0.3	3.7E-01	−0.3 ± 0.2	4.5E-02	−0.5 ± 0.2	2.2E-02	−0.5 ± 0.2	3.6E-02
NAAG	0.2 ± 0.1	1.2E-01	−0.3 ± 0.2	1.5E-01	−0.1 ± 0.2	4.9E-01	0.1 ± 0.2	4.9E-01	0.2 ± 0.2	3.3E-01
NAA+NAAG	−0.07 ± 0.1	6.4E-01	−0.3 ± 0.2	2.3E-01	−0.4 ± 0.2	2.7E-02	−0.4 ± 0.2	2.7E-02	−0.5 ± 0.2	5.2E-02
PE	−0.07 ± 0.1	6.6E-01	0.2 ± 0.3	4.0E-01	0.1 ± 0.2	3.6E-01	0.07 ± 0.2	7.1E-01	0.04 ± 0.2	8.6E-01
Taurine	−0.2 ± 0.1	8.4E-02	−0.2 ± 0.2	4.0E-01	−0.5 ± 0.2	**9.0E-03**	−0.7 ± 0.2	**6.0E-04**	−0.8 ± 0.2	**8.5E-04**
Asp/Glu	0.01 ± 0.2	9.3E-01	−0.4 ± 0.2	7.0E-02	−0.4 ± 0.2	**5.6E-03**	−0.4 ± 0.2	3.2E-02	−0.4 ± 0.2	5.2E-02
Glu/Gln	−0.2 ± 0.1	2.8E-01	0.3 ± 0.2	1.8E-01	0.2 ± 0.2	2.9E-01	0.0 ± 0.2	9.9E-01	−0.06 ± 0.2	7.7E-01
NAA/Ins	−0.2 ± 0.1	5.8E-02	−0.1 ± 0.2	4.9E-01	−0.4 ± 0.1	**2.7E-03**	−0.6 ± 0.1	**3.9E-05**	−0.7 ± 0.1	**1.2E-04**
MM_0.89_	0.08 ± 0.2	5.7E-01	−0.04 ± 0.3	8.7E-01	0.05 ± 0.2	7.5E-01	0.1 ± 0.2	4.6E-01	0.2 ± 0.2	4.6E-01
MM_1.20_	0.2 ± 0.1	1.9E-01	−0.04 ± 0.2	8.5E-01	0.1 ± 0.1	3.1E-01	0.3 ± 0.1	5.7E-02	0.4 ± 0.1	5.9E-02
MM_1.39_	0.1 ± 0.2	5.1E-01	−0.2 ± 0.2	4.2E-01	−0.09 ± 0.2	5.4E-01	0.01 ± 0.2	9.6E-01	0.05 ± 0.2	8.2E-01
MM_1.66_	0.08 ± 0.2	5.7E-01	−0.1 ± 0.2	6.0E-01	−0.03 ± 0.1	8.4E-01	0.06 ± 0.1	7.4E-01	0.09 ± 0.1	6.6E-01
MM_2.02_	−0.2 ± 0.2	3.1E-01	0.4 ± 0.2	1.3E-01	0.2 ± 0.2	1.7E-01	0.05 ± 0.2	7.8E-01	−0.01 ± 0.2	9.5E-01
MM_2.26_	−0.07 ± 0.1	5.9E-01	−0.04 ± 0.2	8.8E-01	−0.1 ± 0.2	4.6E-01	−0.2 ± 0.2	3.0E-01	−0.2 ± 0.2	3.2E-01
MM_2.97_	−0.2 ± 0.1	1.3E-01	0.5 ± 0.2	5.0E-02	0.2 ± 0.1	1.2E-01	0.0 ± 0.1	9.8E-01	−0.09 ± 0.1	6.8E-01
MM_3.84_	−0.2 ± 0.2	3.0E-01	0.4 ± 0.3	1.4E-01	0.2 ± 0.2	1.9E-01	0.04 ± 0.2	8.6E-01	−0.04 ± 0.2	8.8E-01

Linear age-by-genotype interactions and main effects of genotype are shown under age:genotypeTg and genotypeTg (age) columns, respectively. Betas are standardized and SE denotes the standard error of the beta estimate. Unadjusted *P*-values are shown, with bold font denoting P-values that were still significant after applying FDR correction at 5%. The number following each macromolecule denotes the frequency at which the peak is located. Asp/Glu, aspartate/glutamate; Cr + PCr, creatine + phosphocreatine; Glu + Gln, glutamate + glutamine; GPC + PCh, glycerophosphocholine + phosphocholine; GSH, glutathione; MM, macromolecule; NAA/Ins, N-acetylaspartate/myo-inositol; NAA, N-acetylaspartate; NAAG, N-acetylaspartylglutamate; PE, phosphoethanolamine.

A secondary analysis explored a three-way interaction between age, genotype, and sex (Model 4). Both glucose (Glc) and Ins demonstrated three-way interactions but were not significant after FDR correction. Neurochemical trajectories for these metabolites, along with tCho and Gln, which showed sub-threshold (*P* < 0.15) three-way interactions prior to FDR correction, are shown in Fig. 3B. A full summary of linear model results is shown in Supplementary Table 4, with the concentration of each neurochemical (mM) included in Supplementary Table 5. Trajectories of select metabolites are shown in Supplementary Fig. 5.

### The TgF344-AD model displays cognitive impairment by 4 months of age

**Figure 4:**
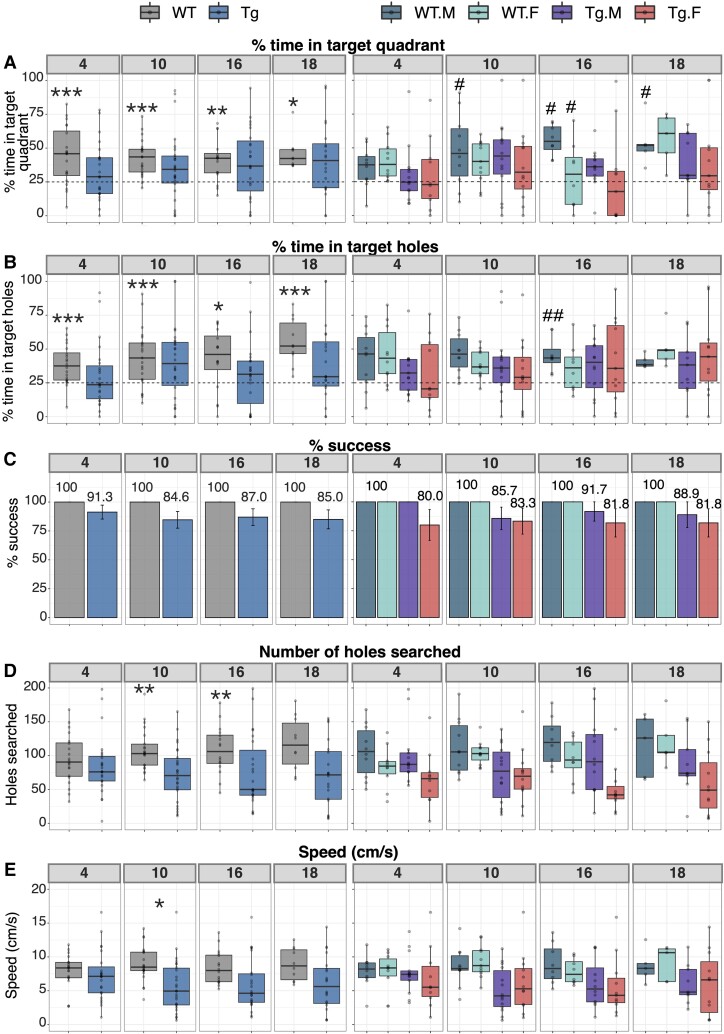
**Barnes Maze probe trial data reveal cognitive impairment as early as 4 months of age**. (**A**) % time spent in the target quadrant and (**B**) % time in target holes were analysed via a one-sample *t*-test for each group against a mean of 25% (chance % of time, indicated by a dotted line), where significance indicates normal cognitive function, and lack of significance indicates impairment. (**C**) % success, (**D**) number of holes searched, and (**E**) speed (cm/s) were analysed via a linear model that included either a main effect of genotype, covarying for sex **(left)**, or an interaction between genotype and sex **(right)**. Bonferroni correction was applied at each time point for data with sex covaried whereby the threshold for significance was ɑ = 0.05/7 = 0.00714. *P < 0.00714, **P < 0.005, ***P < 0.001. Similarly, the threshold for significance for data split by genotype and sex was ɑ = 0.05/11 = 0.00455. #*P* < 0.00455, ##*P* < 0.001. Group sizes per time point for all metrics shown here were 10M/10F WT and 13M/10F Tg at 4 months, 10M/11F WT and 14M/12F Tg at 10 months, 8M/8F WT and 12M/11F Tg at 16 months, and 5M/5F WT and 9M/11F Tg at 18 months. F, female; M, male; Tg, transgenic; WT, wildtype.

Long-term spatial reference memory in Tg and WT rats was evaluated at each time point via the probe trial of the Barnes Maze test, conducted 48 h after the last training trial. Cognitive impairment in Tg rats was evident as early as 4 months of age, as determined by testing the percentage of time WT and Tg rats spent in the target quadrant against the chance amount of time a rat would spend in each quadrant. A mean significantly above 25% is suggestive of intact spatial memory recall, which WT rats demonstrated throughout the study, while Tg rats did not meet the significance threshold at any time points (Fig. 4A (*left*)). A similar effect was seen when testing the percentage of time spent exploring holes within the target quadrant (Fig. 4B (*left*)). WT rats consistently spent more than a chance amount of time exploring holes in the target quadrant, whereas Tg rats did not. Additionally, as shown in Fig. 4C (*left*) the rate of success versus failure in locating the escape hole was lower among Tg rats throughout the study, though this difference was not statistically significant. The number of holes searched and average speed were also measured during the probe trial to characterize level of exploration and mobility, respectively. As shown in Fig. 4D, E (*left*), both metrics differed between WT and Tg rats, with Tgs searching fewer holes throughout the study and moving more slowly than their WT littermates. However, after Bonferroni correction, the differences in holes searched was only significant at 10 and 16 months, while the difference in speed was only significant at 10 months.

A secondary analysis examining the interaction between genotype and sex was also performed. As shown in Fig. 4A (*right*), only WT males consistently spent significantly more than 25% of the time in the target quadrant, although the 4-month time point did not reach the significance threshold after the Bonferroni correction. WT males generally also spent a higher percentage of time in the target holes than WT females, Tg males, or Tg females, but only reached the significance threshold at 16 months (Fig. 4 (*right*)). Interestingly, and as shown in Fig. 4C, D (*right*), Tg females demonstrated the lowest success rate across all time points, and the lowest rates of exploration (fewest holes searched) at three of four time points, though no significant genotype-by-sex interactions were found for either metric. Finally, as seen in Fig. 4E (*right*), no genotype-by-sex interactions were found for speed at any time point. All Barnes Maze statistics can be found in Supplementary Table 6, with summary data for each metric, split by time point, genotype, and sex in Supplementary Table 7.

## Discussion

The TgF344-AD rat is unique in its manifestation of amyloid and tau pathology despite only expressing mutant *APP* and *PS1*, and therefore closely replicates human Alzheimer’s disease. Thorough characterization of the manifestation and progression of physiological abnormalities—particularly those that can be measured non-invasively—comprising each disease stage in this rat model is required for designing effective therapeutic approaches. While other authors have characterized pathological features in this model, the majority of our neuroimaging findings are being reported for the first time and ours is the earliest assessment of cognitive function. This study also explores the intersecting influence of sex and genotype on neuroimaging and behavioural markers, which is crucial for improving diagnostic methods and interventions given that Alzheimer’s disease prevalence and manifestation can differ between men and women.^49^

As determined via post-mortem histology studies, decreased brain volume detected via MRI is an accurate marker of Alzheimer’s disease-related neurodegeneration that can be used to support a clinical diagnosis in humans.^13,50,51^ Models of human disease progression indicate early tissue pathology and atrophy in the entorhinal cortex^52^ and other regions that comprise the limbic system, particularly the hippocampus.^50,53,54^ The TgF344-AD model does not demonstrate significant cortical atrophy by 18 months, but does recapitulate volume reductions in limbic structures, including the hippocampus, basal forebrain, fimbria, fornix, hypothalamus, and lateral septum. In opposition to dentate gyrus atrophy reported in human Alzheimer’s disease,^15,55^ we observed hypertrophy in the TgF344-AD model, with significantly larger volume at 16 and 18 months in Tgs relative to WTs. This is not entirely unexpected given that Fischer 344 rats display increased dentate gyrus volume during normal aging,^56,57^ and therefore further hypertrophy during Alzheimer’s disease may represent a pathological feature, similar to how normal hippocampal atrophy with age is exacerbated in Alzheimer’s disease.^58^ Additional structures with significant atrophy were the nucleus accumbens, caudoputamen, and ventral pallidum, all of which display amyloid and tau pathology and structural changes in human Alzheimer’s disease.^15,54,59^ The only other structural MRI-based study in the TgF344-AD rat reported a lack of major structural differences, but this study used only female rats and therefore a direct comparison between their results and ours is not particularly meaningful.^60^

Age-centred analyses revealed that while many structures atrophied faster in Tgs, this was occasionally preceded by hypertrophy. For example, Tg rats demonstrate larger caudoputamen, fimbria, and nucleus accumbens volumes until 10 months, and then smaller volumes at 16 and 18 months relative to WTs. Supporting these findings, a neuroimaging study in PS1 mutation carriers reported increased caudate volume in asymptomatic individuals but decreased volume in symptomatic individuals,^61^ suggesting different processes underlie morphometric change at different stages of disease progression. Reactive neuronal hypertrophy in the hippocampal CA1 region has been shown in Alzheimer’s disease subjects prior to symptom onset^62^ supporting early regional volume increases, either as a cellular response to amyloid and tau deposition, or a compensatory process prior to degeneration of neurons and synapses.^63^ Future work combining MRI-based volumetric analysis and design-based stereology, similar to studies in transgenic mice,^64,65^ would help fill critical gaps in knowledge regarding mechanisms underlying pathological morphometric change in the TgF344-AD model.

MRS allows for quantification of brain tissue metabolites, providing insight into the biochemical underpinnings of altered brain structure and function.^12^ Similar changes in NAA, Ins, and NAA/Ins to what we report have been shown in the TgF344-AD rat,^66^ the McGill-R-Thy1-APP rat model,^26^ mouse models,^25,67,68^ and in human studies.^27,28,69^ Decreased NAA reflects reduced neuronal viability—specifically mitochondrial dysfunction—as opposed to purely neuronal density.^29,30^ The possible mechanisms behind increased Ins are more varied and may reflect increased glial cell activation and/or inflammation, increased phagocytic activity, or cellular membrane disruption, as Ins is a precursor for inositol lipid synthesis, a constituent of membrane lipids, and an osmolyte.^30,70,71^

Decreased taurine in the TgF344-AD rat is in agreement with the literature supporting the role of taurine in neurite outgrowth, synaptogenesis, and synaptic transmission,^72^ all of which are dysfunctional in Alzheimer’s disease.^73^ Other differences between Tg rats and WT controls included a lower Asp/Glu ratio, lower tCr, and higher Lac at 10 months of age. These differences suggest the TgF344-AD model replicates the well-documented phenomenon of altered bioenergetics in human Alzheimer’s disease,^74,75^ specifically, disrupted excitatory neurotransmission and a shift towards non-oxidative energy metabolism.^29^ These findings also indicate tCr should not be used as an internal reference in this model. Finally, our report of increased tCho is in agreement with studies in human Alzheimer’s disease patients^27,76,77^ and likely reflects increased cell membrane turnover (a feature characteristic of neuronal degeneration^78^) and/or inflammation and astrocytosis.^30,79^ Overall, the neurochemical profile of the TgF344-AD rat closely replicates that of human patients and provides insight into numerous pathological processes, substantiating its application in Alzheimer’s disease research. In particular, our findings build upon the study in TgF344-AD rats by Chaney *et al*.,^66^ by studying both male and female rats, characterizing additional metabolites, and through the inclusion of individual macromolecule peaks, which, while they did not differ between WT and TgF344-AD rats, inclusion of basis functions for these peaks as performed here has been shown to improve metabolite quantification. ^80,81^

Deficits in hippocampus-dependent spatial learning and memory are among the earliest complaints in Alzheimer’s disease subjects.^82–84^ Previous studies indicate that 5-month-old TgF344-AD rats require more trials to learn a delayed non-match-to-sample task^85^ and impaired reversal learning on the Morris Water Maze^86^ and Barnes Maze^10^ by 6 months. To test spatial navigation in our TgF344-AD rats, we used a shortened version of the Barnes Maze test^44^ which detected impairment in 3xTg mice earlier than traditional protocols.^43^ Fittingly, ours is the earliest report of cognitive disturbance in this model, with impairments in long-term spatial reference memory present by 4 months of age. We also noted genotype-dependent differences in speed and number of holes searched, substantiating the choice to use speed- and motivation-independent measures rather than the frequently chosen escape latency or number of errors.^87,88^

Regarding the interaction between sex and genotype, Tg females demonstrated stronger pathological effects in several brain volumes and more cognitive decline than Tg males, whereas sex effects on metabolite concentration were also present but did not consistently impact Tg females. To our knowledge, we are the first group to examine the interaction between age, genotype, and sex on neuroimaging markers in this model. However, sex differences in the TgF344-AD rat exist in open field and buried food tasks^89^ and the Morris Water Maze test,^90^ and sex differences in neuroanatomy and hippocampal tissue chemistry during normal aging in the Fischer 344 rat have been reported.^32,37^ These findings generally recapitulate human Alzheimer’s disease data. Sex-specific patterns of neurodegeneration exist in human patients^91,92^ and men frequently present with later and less severe cognitive deficits than women.^93,94^ There is also an established role for oestrogen in regulation of metabolic pathways affected by Alzheimer’s disease such as Glc transport, aerobic glycolysis, and mitochondrial function.^73,94,95^ While additional work is required to corroborate our findings, the TgF344-AD model appears to recapitulate known sex differences in several aspects of disease presentation.

Regarding corroboration of the timing of pathological changes that we report in the TgF344-AD rat, previous studies show by 5–6 months of age, TgF344-AD rats display disruption in hippocampal-dependent synaptic circuits,^85,96^ dysfunction of the noradrenergic system,^86^ and loss of functional connectivity prior to the appearance of microstructural alterations.^60^ Reduced maximum synaptic transmission in the hippocampus occurs between 9 and 12 months, in the absence of reduced dendritic spine density,^97^ continuing to support a timeline of functional change prior to significant morphometric change. Profound cerebral microvascular and neuronal network dysfunction is present at 9 months,^98,99^ along with reduced antioxidant capacity, and increased reactive oxygen species and pro-inflammatory cytokines at 10 months.^100^ Our report of neurochemical changes by 10 months is consistent with these previously described molecular events. At 13 months, TgF344-AD rats demonstrate deficits in hippocampal neuronal differentiation, migration, and survival,^101^ and display significant tau pathology, neuronal damage, and cognitive impairment between 16 and 26 months.^10,66,102^ Given that reduced synaptic density and neuronal loss are associated with MRI-detectable volume changes,^51,103^ these reports of altered neurogenesis and neuronal damage may reflect some of the processes underlying the volumetric changes we report.

This timeline of biochemical changes preceding substantive structural abnormalities is corroborated by models of disease progression^3^ and studies exploring upstream and downstream processes of amyloid and tau deposition.^63,73,104^ Additionally, the timing of biochemical and structural changes around midlife to the beginning of senescence suggests altered neurochemistry and neuroanatomy may be in response to amyloid and tau pathological load,^10,86^ and are not evident before the appearance of gross tissue pathology. In contrast, early cognitive impairment differs from disease progression in humans where cognitive complaints are among the last pathological features to manifest. Given numerous studies have validated the consistency with which this model mimics the spread of components of human Alzheimer’s pathology, this difference in timing of cognitive dysfunction may reflect inconsistencies in how cognition is tested or presents in rodent models versus humans. Behavioural testing is also extremely variable and subjective. Neuroimaging is considerably more objective and less variable, thus providing a better powered, sensitive, accurate, and efficient means to characterize disease progression. To our knowledge, we are the first group to simultaneously characterize the timing of the manifestation of neuroanatomical, neurochemical, and cognitive abnormalities in the TgF344-AD rat, thus providing new information that can be used to define disease stages in this model.

There are limitations to consider when interpreting the results of the present study. First, this study does not include any histological data, limiting our ability to understand the cellular underpinnings of the neurochemical, neuroanatomical, and cognitive changes that we report. Thorough characterization of tissue pathological load of disease hallmarks in the TgF344-AD model has been performed by other authors, confirming the presence of amyloid-β deposition, gliosis, neuronal loss, and tauopathy^10,66,100^ in regions and along a time course similar to that of human Alzheimer’s disease.^79^ A future study combining *in vivo* neuroimaging with *ex vivo* histological analyses, particularly examining both number and morphology of microglia, astrocytes, and neurons would greatly contribute to our knowledge regarding the cellular basis for the changes in neuroimaging markers that we report here.

Second, the use of a polynomial age term in the volumetric analyses, which was necessary given the non-linear change with age that we and others report,^19,56,105,106^ likely reduced our power to detetct age-by-genotype-by-sex interactions. Given that most structures demonstrated volume change towards 16 months, a paradigm where brain volumes are quantified from midlife onwards may permit the use of a linear age term and provide more power to detect three-way interactions. Second, restrictions on facility access due to the COVID-19 pandemic resulted in fewer animals being tested and increased variation in testing dates at the final time point, as well as inconsistency in the time between staining and imaging during immunofluorescence experiments. While these inconsistencies were accounted for in the statistical modelling, this is likely to have increased the overall variation in the data, possibly masking or muting some of the effects at the final time point. Finally, the lack of histological analyses at early time points meant we were unable to determine if pathological changes in neuroimaging markers precede those at the cellular level in the TgF344-AD rat. This gap in knowledge limits our ability to interpret the origin of the altered neuroimaging and cognitive markers that we report.

Altogether, our results provide a comprehensive review of multiple phenotypic components of pathology in the TgF344-AD model, characterized from early to late stages of disease progression. This longitudinal multimodal study demonstrates that the TgF344-AD rat recapitulates major neurochemical, neuroanatomical, and cognitive features of human Alzheimer’s disease, and furthers our understanding of the many processes comprising disease progression. These findings support the use of MRI and MRS to monitor disease progression in rodent models of Alzheimer’s disease *in vivo and* contribute to the growing body of work validating the TgF344-AD rat as a highly relevant model of Alzheimer’s disease.

## Supplementary Material

fcac072_Supplementary_Data

## Abbreviations

APP =: amyloid precursor protein
Asp =: aspartate
FDR =: false discovery rate
Glc =: glucose
Gln =: glutamine
GM =: grey matter
Glu =: glutamate
Ins =: myo-inositol
Lac =: lactate
MRS =: magnetic resonance spectroscopy
NAA =: N-acetylaspartate
PS =: presenilin
tCho =: total choline
tCr =: total creatine
Tg =: transgenic
WM =: white matter
WT =: wildtype

## References

[1] 2020 Alzheimer’s disease facts and figures . Alzheimers Dement 2020;16(3)391–460. 10.1002/alz.12068 32157811

[2] Jr JC , Knopman DS, Jagust WJ, et al Hypothetical model of dynamic biomarkers of the Alzheimer’s pathological cascade. Lancet Neurol 2010;9(1):119–128. 20083042 10.1016/S1474-4422(09)70299-6 PMC2819840

[3] Jr JC , Knopman DS, Jagust WJ, et al Tracking pathophysiological processes in Alzheimer’s disease: an updated hypothetical model of dynamic biomarkers. Lancet Neurol 2013;12(2):207–216. 23332364 10.1016/S1474-4422(12)70291-0 PMC3622225

[4] Bateman RJ , Xiong C, Benzinger TLS, et al Clinical and biomarker changes in dominantly inherited Alzheimer’s disease. N Engl J Med 2012;367(9):795–804. 22784036 10.1056/NEJMoa1202753 PMC3474597

[5] Drummond E , Wisniewski T. Alzheimer’s disease: Experimental models and reality. Acta Neuropathol 2017;133(2):155–175. 28025715 10.1007/s00401-016-1662-x PMC5253109

[6] Carmo S D , Cuello AC. Modeling Alzheimer’s disease in transgenic rats. Mol Neurodegener 2013;8(37). 10.1186/1750-1326-8-37 PMC4231465 24161192

[7] Selkoe DJ . Alzheimer’s disease. Cold Spring Harb Perspect Biol 2011;3(7):a004457. 21576255 10.1101/cshperspect.a004457 PMC3119915

[8] Lewis J , Dickson DW, Lin WL, et al Enhanced neurofibrillary degeneration in transgenic mice expressing mutant tau and APP. Science 2001;293(5534):1487–1491. 11520987 10.1126/science.1058189

[9] Oddo S , Caccamo A, Shepherd JD, et al Triple-transgenic model of Alzheimer’s disease with plaques and tangles: intracellular Abeta and synaptic dysfunction. Neuron 2003;39(3):409–421. 12895417 10.1016/s0896-6273(03)00434-3

[10] Cohen RM , Rezai-Zadeh K, Weitz TM, et al A transgenic Alzheimer rat with plaques, tau pathology, behavioral impairment, oligomeric aβ, and frank neuronal loss. J Neurosci 2013;33(15):6245–6256. 23575824 10.1523/JNEUROSCI.3672-12.2013 PMC3720142

[11] Ellenbroek B , Youn J. Rodent models in neuroscience research: Is it a rat race? Dis Model Mech 2016;9(10):1079–1087. 27736744 10.1242/dmm.026120 PMC5087838

[12] Gao F , Barker PB. Various MRS application tools for Alzheimer disease and mild cognitive impairment. AJNR Am J Neuroradiol 2014;35(6 Suppl):S4–S11. 24742809 10.3174/ajnr.A3944 PMC4401041

[13] Frisoni GB , Fox NC, Jr JC, Scheltens P, Thompson PM. The clinical use of structural MRI in Alzheimer disease. Nat Rev Neurol 2010;6(2):67–77. 20139996 10.1038/nrneurol.2009.215 PMC2938772

[14] Mueller SG , Schuff N, Weiner MW. Evaluation of treatment effects in Alzheimer’s and other neurodegenerative diseases by MRI and MRS. NMR Biomed 2006;19(6):655–668. 16986115 10.1002/nbm.1062 PMC1820857

[15] Pini L , Pievani M, Bocchetta M, et al Brain atrophy in Alzheimer’s Disease and aging. Ageing Res Rev 2016;30:25–48. 26827786 10.1016/j.arr.2016.01.002

[16] Jr JC , Shiung MM, Weigand SD, et al Brain atrophy rates predict subsequent clinical conversion in normal elderly and amnestic MCI. Neurology 2005;65(8):1227–1231. 16247049 10.1212/01.wnl.0000180958.22678.91 PMC2753547

[17] van de Pol LA , van der Flier WM, Korf ESC, Fox NC, Barkhof F, Scheltens P. Baseline predictors of rates of hippocampal atrophy in mild cognitive impairment. Neurology 2007;69(15):1491–1497. 17923611 10.1212/01.wnl.0000277458.26846.96

[18] Lau JC , Lerch JP, Sled JG, Henkelman RM, Evans AC, Bedell BJ. Longitudinal neuroanatomical changes determined by deformation-based morphometry in a mouse model of Alzheimer’s disease. Neuroimage 2008;42(1):19–27. 18547819 10.1016/j.neuroimage.2008.04.252

[19] Kong V , Devenyi GA, Gallino D, et al Early-in-life neuroanatomical and behavioural trajectories in a triple transgenic model of Alzheimer’s disease. Brain Struct Funct 2018;223(7):3365–3382. 29948190 10.1007/s00429-018-1691-4

[20] Badhwar A , Lerch JP, Hamel E, Sled JG. Impaired structural correlates of memory in Alzheimer’s disease mice. Neuroimage Clin 2013;3:290–300. 24273714 10.1016/j.nicl.2013.08.017 PMC3814975

[21] Spencer NG , Bridges LR, Elderfield K, Amir K, Austen B, Howe FA. Quantitative evaluation of MRI and histological characteristics of the 5xFAD Alzheimer mouse brain. Neuroimage 2013;76:108–115. 23507393 10.1016/j.neuroimage.2013.02.071

[22] Maheswaran S , Barjat H, Rueckert D, et al Longitudinal regional brain volume changes quantified in normal aging and Alzheimer’s APP x PS1 mice using MRI. Brain Res 2009;1270:19–32. 19272356 10.1016/j.brainres.2009.02.045

[23] Dedeoglu A , Choi J-K, Cormier K, Kowall NW, Jenkins BG. Magnetic resonance spectroscopic analysis of Alzheimer’s disease mouse brain that express mutant human APP shows altered neurochemical profile. Brain Res 2004;1012(1-2):60–65. 15158161 10.1016/j.brainres.2004.02.079

[24] Choi J-K , Carreras I, Aytan N, Jenkins-Sahlin E, Dedeoglu A, Jenkins BG. The effects of aging, housing and ibuprofen treatment on brain neurochemistry in a triple transgene Alzheimer’s disease mouse model using magnetic resonance spectroscopy and imaging. Brain Res 2014;1590:85–96. 25301691 10.1016/j.brainres.2014.09.067 PMC4274938

[25] Marjanska M , Curran GL, Wengenack TM, et al Monitoring disease progression in transgenic mouse models of Alzheimer’s disease with proton magnetic resonance spectroscopy. Proc Natl Acad Sci U S A 2005;102(33):11906–11910. 16091461 10.1073/pnas.0505513102 PMC1188012

[26] Nilsen LH , Melø TM, Saether O, Witter MP, Sonnewald U. Altered neurochemical profile in the McGill-R-Thy1-APP rat model of Alzheimer’s disease: a longitudinal in vivo 1 H MRS study. J Neurochem 2012;123(4):532–541. 22943908 10.1111/jnc.12003

[27] Marjańska M , McCarten JR, Hodges JS, Hemmy LS, Terpstra M. Distinctive neurochemistry in Alzheimer’s disease via 7 T in vivo magnetic resonance spectroscopy. J Alzheimers Dis 2019;68(2):559–569. 30775983 10.3233/JAD-180861 PMC6481537

[28] Murray ME , Przybelski SA, Lesnick TG, et al Early Alzheimer’s disease neuropathology detected by proton MR spectroscopy. J Neurosci 2014;34(49):16247–16255. 25471565 10.1523/JNEUROSCI.2027-14.2014 PMC4252542

[29] McKenna MC , Dienel GA, Sonnewald U, Waagepetersen HS, Schousboe A. Chapter 11–Energy Metabolism of the Brain. In: Brady ST, Siegel GJ, Albers RW, Price DL, eds. Basic Neurochemistry. 8th edn. Academic Press; 2012:200–231.

[30] Ross AJ , Sachdev PS. Magnetic resonance spectroscopy in cognitive research. Brain Res Brain Res Rev 2004;44(2-3):83–102. 15003387 10.1016/j.brainresrev.2003.11.001

[31] Lerch JP , Gazdzinski L, Germann J, Sled JG, Henkelman RM, Nieman BJ. Wanted dead or alive? The tradeoff between in-vivo versus ex-vivo MR brain imaging in the mouse. Front Neuroinform 2012;6:6. 22470335 10.3389/fninf.2012.00006 PMC3311228

[32] Fowler C , Goerzen D, Madularu D, Devenyi GA, Chakravarty MM, Near J. Longitudinal characterization of neuroanatomical changes in the Fischer 344 rat brain during normal aging and between sexes. Neurobiol Aging 2021;109:216–228. 34775212 10.1016/j.neurobiolaging.2021.10.003

[33] Goerzen D , Fowler C, Devenyi GA, et al An MRI-derived neuroanatomical atlas of the Fischer 344 rat brain. Sci Rep 2020;10(1):6952. 32332821 10.1038/s41598-020-63965-x PMC7181609

[34] Friedel M , van Eede MC, Pipitone J, Chakravarty MM, Lerch JP. Pydpiper: A flexible toolkit for constructing novel registration pipelines. Front Neuroinform 2014;8:67. 25126069 10.3389/fninf.2014.00067 PMC4115634

[35] Chung MK , Worsley KJ, Paus T, et al A unified statistical approach to deformation-based morphometry. Neuroimage 2001;14(3):595–606. 11506533 10.1006/nimg.2001.0862

[36] Lerch J , Hammill C, van Eede M, Cassel D. Statistical Tools for Medical Imaging NetCDF (MINC) Files. R package version 1.5.2.3. Published 2017. http://mouse-imaging-centre.github.io/RMINC/

[37] Fowler CF , Madularu D, Dehghani M, Devenyi GA, Near J. Longitudinal quantification of metabolites and macromolecules reveals age-and sex-related changes in the healthy Fischer 344 rat brain. Neurobiol Aging 2020;101:109–122. 33610061 10.1016/j.neurobiolaging.2020.12.012

[38] Automatic GR . Localized in vivo adjustment of all first- and second-order shim coils. J Magn Reson Med 1993;29:804–811. 10.1002/mrm.1910290613 8350724

[39] Tkáč I , Starčuk Z-Y, Choi I, Gruetter R. In VivoH NMR Spectroscopy of Rat Brain at 1 ms Echo Time. Magn Reson Med. 1999; 41:649–656. 10332839 10.1002/(sici)1522-2594(199904)41:4<649::aid-mrm2>3.0.co;2-g

[40] Simpson R , Devenyi GA, Jezzard P, Hennessy TJ, Near J. Advanced processing and simulation of MRS data using the FID appliance (FID-A)-An open source, MATLAB-based toolkit. Magn Reson Med 2017;77(1):23–33. 26715192 10.1002/mrm.26091

[41] Provencher SW . Automatic quantitation of localized in vivo 1H spectra with LCModel. NMR Biomed 2001;14(4):260–264. 11410943 10.1002/nbm.698

[42] Ernst T , Kreis R, Ross BD. Absolute quantitation of water and metabolites in the human brain. I. Compartments and Water. J Magn Reson B 1993;102(1):1–8.

[43] Attar A , Liu T, Chan W-TC, et al A shortened Barnes maze protocol reveals memory deficits at 4-months of age in the triple-transgenic mouse model of Alzheimer’s disease. PLoS One 2013;8(11):e80355. 24236177 10.1371/journal.pone.0080355 PMC3827415

[44] Barnes CA . Memory deficits associated with senescence: A neurophysiological and behavioral study in the rat. J Comp Physiol Psychol 1979;93(1):74–104. 221551 10.1037/h0077579

[45] R Core Team . R: A Language and Environment for Statistical Computing. Published online 2020. https://www.R-project.org/

[46] Bernal-Rusiel JL , Greve DN, Reuter M, Fischl B, Sabuncu MR. Alzheimer’s disease neuroimaging initiative. Statistical analysis of longitudinal neuroimage data with Linear Mixed Effects models. Neuroimage 2013;66:249–260. 23123680 10.1016/j.neuroimage.2012.10.065 PMC3586747

[47] Bartha R . Effect of signal-to-noise ratio and spectral linewidth on metabolite quantification at 4 T. NMR Biomed 2007;20(5):512–521. 17205487 10.1002/nbm.1122

[48] Benjamini Y , Hochberg Y. Controlling the false discovery rate: A practical and powerful approach to multiple testing. J R Stat Soc Series B Stat Methodol 1995;57(1):289–300.

[49] Mazure CM , Swendsen J. Sex differences in Alzheimer’s disease and other dementias. Lancet Neurol 2016;15(5):451–452. 26987699 10.1016/S1474-4422(16)00067-3 PMC4864429

[50] Jack CR , Barkhof F, Bernstein MA, et al Steps to standardization and validation of hippocampal volumetry as a biomarker in clinical trials and diagnostic criterion for Alzheimer’s disease. Alzheimers Dement 2011;7(4):474–485.e4. 21784356 10.1016/j.jalz.2011.04.007 PMC3396131

[51] Bobinski M , de Leon MJ, Wegiel J, et al The histological validation of post mortem magnetic resonance imaging-determined hippocampal volume in Alzheimer’s disease. Neuroscience 1999;95(3):721–725. 10.1016/s0306-4522(99)00476-5 10670438

[52] Braak H , Braak E. Staging of Alzheimer’s disease-related neurofibrillary changes. Neurobiol Aging 1995;16(3):271–278. 7566337 10.1016/0197-4580(95)00021-6

[53] Callen DJ , Black SE, Gao F, Caldwell CB, Szalai JP. Beyond the hippocampus: MRI volumetry confirms widespread limbic atrophy in AD. Neurology 2001;57(9):1669–1674. 11706109 10.1212/wnl.57.9.1669

[54] Braak H , Braak E. Alzheimer’s disease affects limbic nuclei of the thalamus. Acta Neuropathol 1991;81(3):261–268. 1711755 10.1007/BF00305867

[55] Wisse LEM , Biessels GJ, Heringa SM, et al Hippocampal subfield volumes at 7T in early Alzheimer’s disease and normal aging. Neurobiol Aging 2014;35(9):2039–2045. 24684788 10.1016/j.neurobiolaging.2014.02.021

[56] Fowler C , Goerzen D, Madularu D, Devenyi GA, Mallar Chakravarty M, Near J. Longitudinal characterization of neuroanatomical changes in the Fischer 344 rat brain during normal aging and between sexes. bioRxiv. Published online April 13, 2021:2021.04.12.439510. 10.1016/j.neurobiolaging.2021.10.003 34775212

[57] Alexander GE , Lin L, Yoshimaru ES, et al Age-related regional network covariance of magnetic resonance imaging gray matter in the rat. Front Aging Neurosci 2020;12:267. 33005147 10.3389/fnagi.2020.00267 PMC7479213

[58] Fjell AM , McEvoy L, Holland D, Dale AM, Walhovd KB. Alzheimer’s Disease Neuroimaging Initiative. What is normal in normal aging? Effects of aging, amyloid and Alzheimer’s disease on the cerebral cortex and the hippocampus. Prog Neurobiol 2014;117:20–40. 24548606 10.1016/j.pneurobio.2014.02.004 PMC4343307

[59] de Jong LW , van der Hiele K, Veer IM, et al Strongly reduced volumes of putamen and thalamus in Alzheimer’s disease: an MRI study. Brain 2008;131(Pt 12):3277–3285. 19022861 10.1093/brain/awn278 PMC2639208

[60] Anckaerts C , Blockx I, Summer P, et al Early functional connectivity deficits and progressive microstructural alterations in the TgF344-AD rat model of Alzheimer’s Disease: A longitudinal MRI study. Neurobiol Dis 2019;124:93–107. 30445024 10.1016/j.nbd.2018.11.010

[61] Fortea J , Sala-Llonch R, Bartrés-Faz D, et al Increased cortical thickness and caudate volume precede atrophy in PSEN1 mutation carriers. J Alzheimers Dis 2010;22(3):909–922. 20858974 10.3233/JAD-2010-100678

[62] Riudavets MA , Iacono D, Resnick SM, et al Resistance to Alzheimer’s pathology is associated with nuclear hypertrophy in neurons. Neurobiol Aging 2007;28(10):1484–1492. 17599696 10.1016/j.neurobiolaging.2007.05.005 PMC2694127

[63] Mattson MP . Pathways towards and away from Alzheimer’s disease. Nature 2004;430(7000):631–639. 15295589 10.1038/nature02621 PMC3091392

[64] West MJ , Bach G, Søderman A, Jensen JL. Synaptic contact number and size in stratum radiatum CA1 of APP/PS1DeltaE9 transgenic mice. Neurobiol Aging 2009;30(11):1756–1776. 18336954 10.1016/j.neurobiolaging.2008.01.009

[65] Oh ES , Savonenko AV, King JF, et al Amyloid precursor protein increases cortical neuron size in transgenic mice. Neurobiol Aging 2009;30(8):1238–1244. 18304698 10.1016/j.neurobiolaging.2007.12.024 PMC2796369

[66] Chaney AM , Lopez-Picon FR, Serrière S, et al Prodromal neuroinflammatory, cholinergic and metabolite dysfunction detected by PET and MRS in the TgF344-AD transgenic rat model of AD: a collaborative multi-modal study. Theranostics 2021;11(14):6644–6667. 34093845 10.7150/thno.56059 PMC8171096

[67] Oberg J , Spenger C, Wang F-H, et al Age related changes in brain metabolites observed by 1H MRS in APP/PS1 mice. Neurobiol Aging 2008;29(9):1423–1433. 17434239 10.1016/j.neurobiolaging.2007.03.002

[68] Güell-Bosch J , Lope-Piedrafita S, Esquerda-Canals G, Montoliu-Gaya L, Villegas S. Progression of Alzheimer’s disease and effect of scFv-h3D6 immunotherapy in the 3xTg-AD mouse model: An in vivo longitudinal study using Magnetic Resonance Imaging and Spectroscopy. NMR Biomed 2020;33(5):e4263. 32067292 10.1002/nbm.4263

[69] Wang H , Tan L, Wang H-F, et al Magnetic resonance spectroscopy in Alzheimer’s Disease: Systematic review and meta-analysis. J Alzheimers Dis 2015;46(4):1049–1070. 26402632 10.3233/JAD-143225

[70] Best JG , Stagg CJ, Dennis A. Chapter 2.5–Other Significant Metabolites: Myo-Inositol, GABA, Glutamine, and Lactate. In: Stagg C and Rothman D, eds. Magnetic Resonance Spectroscopy. Academic Press; 2014:122–138.

[71] Brand A , Richter-Landsberg C, Leibfritz D. Multinuclear NMR studies on the energy metabolism of glial and neuronal cells. Dev Neurosci 1993;15:289–298. 7805581 10.1159/000111347

[72] Mersman B , Zaidi W, Syed NI, Xu F. Taurine promotes neurite outgrowth and synapse development of both vertebrate and invertebrate central neurons. Front Synaptic Neurosci 2020;12:29. 32792935 10.3389/fnsyn.2020.00029 PMC7387692

[73] Camandola S , Mattson MP. Brain metabolism in health, aging, and neurodegeneration. EMBO J 2017;36(11):1474–1492. 28438892 10.15252/embj.201695810 PMC5452017

[74] Yin F , Sancheti H, Patil I, Cadenas E. Energy metabolism and inflammation in brain aging and Alzheimer’s disease. Free Radic Biol Med 2016;100:108–122. 27154981 10.1016/j.freeradbiomed.2016.04.200 PMC5094909

[75] Mosconi L . Glucose metabolism in normal aging and Alzheimer’s disease: Methodological and physiological considerations for PET studies. Clin Transl Imaging 2013;1(4):217–233. 10.1007/s40336-013-0026-y PMC3881550 24409422

[76] Pfefferbaum A , Adalsteinsson E, Spielman D, Sullivan EV, Lim KO. In vivo brain concentrations of N-acetyl compounds, creatine, and choline in Alzheimer disease. Arch Gen Psychiatry 1999;56(2):185–192. 10025444 10.1001/archpsyc.56.2.185

[77] Kantarci K , Petersen RC, Boeve BF, et al 1H MR spectroscopy in common dementias. Neurology 2004;63(8):1393–1398. 15505154 10.1212/01.wnl.0000141849.21256.ac PMC2766798

[78] Lin JC , Gant N. Chapter 2.3–The Biochemistry of Choline. In: Stagg C, Rothman D, eds. Magnetic Resonance Spectroscopy. Academic Press; 2014:104–110.

[79] Selkoe DJ . Alzheimer’s disease: Genes, proteins, and therapy. Physiol Rev 2001;81(2):741–766. 11274343 10.1152/physrev.2001.81.2.741

[80] Hofmann L , Slotboom J, Jung B, Maloca P, Boesch C, Kreis R. Quantitative 1H-magnetic resonance spectroscopy of human brain: Influence of composition and parameterization of the basis set in linear combination model-fitting. Magn Reson Med 2002;48(3):440–453. 12210908 10.1002/mrm.10246

[81] Cudalbu C , Mlynárik V, Gruetter R. Handling macromolecule signals in the quantification of the neurochemical profile. J Alzheimers Dis 2012;31(Suppl 3):S101–S115. 22543852 10.3233/JAD-2012-120100

[82] Chan D , Gallaher LM, Moodley K, Minati L, Burgess N, Hartley T. The 4 mountains test: A short test of spatial memory with high sensitivity for the diagnosis of pre-dementia Alzheimer’s disease. J Vis Exp 2016(116):54454. 10.3791/54454 PMC5092189 27768046

[83] Bianchini F , Di Vita A, Palermo L, Piccardi L, Blundo C, Guariglia C. A selective egocentric topographical working memory deficit in the early stages of Alzheimer’s disease: a preliminary study. Am J Alzheimers Dis Other Demen 2014;29(8):749–754. 24906969 10.1177/1533317514536597 PMC10852801

[84] Lithfous S , Dufour A, Després O. Spatial navigation in normal aging and the prodromal stage of Alzheimer’s disease: insights from imaging and behavioral studies. Ageing Res Rev 2013;12(1):201–213. 22771718 10.1016/j.arr.2012.04.007

[85] Muñoz-Moreno E , Tudela R, López-Gil X, Soria G. Early brain connectivity alterations and cognitive impairment in a rat model of Alzheimer’s disease. Alzheimers Res Ther 2018;10(1):16. 29415770 10.1186/s13195-018-0346-2 PMC5803915

[86] Rorabaugh JM , Chalermpalanupap T, Botz-Zapp CA, et al Chemogenetic locus coeruleus activation restores reversal learning in a rat model of Alzheimer’s disease. Brain 2017;140(11):3023–3038. 29053824 10.1093/brain/awx232 PMC5841201

[87] Gawel K , Gibula E, Marszalek-Grabska M, Filarowska J, Kotlinska JH. Assessment of spatial learning and memory in the Barnes maze task in rodents-methodological consideration. Naunyn Schmiedebergs Arch Pharmacol 2019;392(1):1–18. 10.1007/s00210-018-1589-y PMC6311199 30470917

[88] Pitts MW . Barnes maze procedure for spatial learning and memory in mice. Bio Protoc 2018;8(5):e2744. 10.21769/BioProtoc.2744 PMC5891830 29651452

[89] Saré RM , Cooke SK, Krych L, Zerfas PM, Cohen RM, Smith CB. Behavioral phenotype in the TgF344-AD rat model of Alzheimer’s disease. Front Neurosci 2020;14:601. 32612506 10.3389/fnins.2020.00601 PMC7308710

[90] Berkowitz LE , Harvey RE, Drake E, Thompson SM, Clark BJ. Progressive impairment of directional and spatially precise trajectories by TgF344-Alzheimer’s disease rats in the Morris Water Task. Sci Rep 2018;8(1):16153. 30385825 10.1038/s41598-018-34368-w PMC6212523

[91] Podcasy JL , Epperson CN. Considering sex and gender in Alzheimer disease and other dementias. Dialogues Clin Neurosci 2016;18(4):437–446. 28179815 10.31887/DCNS.2016.18.4/cepperson PMC5286729

[92] Martínez-Pinilla E , Ordóñez C, Del Valle E, Navarro A, Tolivia J. Regional and gender study of neuronal density in brain during aging and in Alzheimer’s disease. Front Aging Neurosci 2016;8:213. 27679571 10.3389/fnagi.2016.00213 PMC5020132

[93] Filon JR , Intorcia AJ, Sue LI, et al Gender differences in Alzheimer disease: brain atrophy, histopathology burden, and cognition. J Neuropathol Exp Neurol 2016;75(8):748–754. 27297671 10.1093/jnen/nlw047 PMC7299435

[94] Rahman A , Jackson H, Hristov H, et al Sex and gender driven modifiers of Alzheimer’s: The role for estrogenic control across age, race, medical, and lifestyle risks. Front Aging Neurosci 2019;11:315. 31803046 10.3389/fnagi.2019.00315 PMC6872493

[95] Rettberg JR , Yao J, Brinton RD. Estrogen: a master regulator of bioenergetic systems in the brain and body. Front Neuroendocrinol 2014;35(1):8–30. 23994581 10.1016/j.yfrne.2013.08.001 PMC4024050

[96] Stoiljkovic M , Kelley C, Stutz B, Horvath TL, Hajós M. Altered cortical and hippocampal excitability in TgF344-AD rats modeling Alzheimer’s disease pathology. Cereb Cortex 2019;29(6):2716–2727. 29920597 10.1093/cercor/bhy140 PMC7302691

[97] Smith LA , McMahon LL. Deficits in synaptic function occur at medial perforant path-dentate granule cell synapses prior to Schaffer collateral-CA1 pyramidal cell synapses in the novel TgF344-Alzheimer’s Disease Rat Model. Neurobiol Dis 2018;110:166–179. 29199135 10.1016/j.nbd.2017.11.014 PMC6661255

[98] Joo IL , Lai AY, Bazzigaluppi P, et al Early neurovascular dysfunction in a transgenic rat model of Alzheimer’s disease. Sci Rep 2017;7(1):1–14. 28401931 10.1038/srep46427 PMC5388880

[99] Bazzigaluppi P , Beckett TL, Koletar MM, et al Early-stage attenuation of phase-amplitude coupling in the hippocampus and medial prefrontal cortex in a transgenic rat model of Alzheimer’s disease. J Neurochem 2018;144(5):669–679. 28777881 10.1111/jnc.14136

[100] Wu C , Yang L, Li Y, et al Effects of exercise training on anxious-depressive-like behavior in Alzheimer rat. Med Sci Sports Exerc 2020;52(7):1456–1469. 32028456 10.1249/MSS.0000000000002294 PMC8015320

[101] Morrone CD , Bazzigaluppi P, Beckett TL, et al Regional differences in Alzheimer’s disease pathology confound behavioural rescue after amyloid-β attenuation. Brain 2020;143(1):359–373. 31782760 10.1093/brain/awz371 PMC6935751

[102] Voorhees JR , Remy MT, Cintrón-Pérez CJ, et al (-)-P7C3-S243 protects a rat model of Alzheimer’s disease from neuropsychiatric deficits and neurodegeneration without altering amyloid deposition or reactive glia. Biol Psychiatry 2018;84(7):488–498. 29246437 10.1016/j.biopsych.2017.10.023 PMC6415524

[103] Apostolova LG , Zarow C, Biado K, et al Relationship between hippocampal atrophy and neuropathology markers: a 7T MRI validation study of the EADC-ADNI Harmonized Hippocampal Segmentation Protocol. Alzheimers Dement 2015;11(2):139–150. 25620800 10.1016/j.jalz.2015.01.001 PMC4348340

[104] Mattson MP , Arumugam TV. Hallmarks of brain aging: Adaptive and pathological modification by metabolic states. Cell Metab 2018;27(6):1176–1199. 29874566 10.1016/j.cmet.2018.05.011 PMC6039826

[105] Pfefferbaum A , Rohlfing T, Rosenbloom MJ, Chu W, Colrain IM, Sullivan EV. Variation in longitudinal trajectories of regional brain volumes of healthy men and women (ages 10 to 85 years) measured with atlas-based parcellation of MRI. Neuroimage 2013;65:176–193. 23063452 10.1016/j.neuroimage.2012.10.008 PMC3516371

[106] Tullo S , Patel R, Devenyi GA, et al MR-based age-related effects on the striatum, globus pallidus, and thalamus in healthy individuals across the adult lifespan. Hum Brain Mapp 2019;40(18):5269–5288. 31452289 10.1002/hbm.24771 PMC6864890

