# Supplementary material for: Neurochemical and cognitive changes precede structural abnormalities in the TgF344-AD rat model
Source: Brain Commun. 2022 Mar 25;4(2):fcac072. doi: 10.1093/braincomms/fcac072 (PMC9007326; doi:10.1093/braincomms/fcac072)
Supplement: fcac072_Supplementary_Data [file fcac072_supplementary_data.zip › Supplementary__material.pdf]

Supplementary Material

Supplementary Methods: additional methodological information for sample size calculations/group sizes, MRI, MRS, Barnes Maze, and statistical analysis

Sample Size Calculations and Group Sizes

The primary hypothesis for this study involved characterizing differences between Tg and WT rats while controlling for sex. Based on test-retest experiments performed in our lab examining intra- and inter-subject variation in hippocampal volume, this study was designed to have 80% power to detect an effect size of 3% with data collected from n=17 animals per genotype over four time points with an estimated type-1 error rate of less than 0.05.[1] Test-retest results for major metabolites of interest (NAA, Ins, tCr, Glu, Gln, tCho) were comparable to those obtained for hippocampal volume and thus the power simulation above generally applies to the main metabolites quantified here. Group sizes were increased from 17 to 24 and 26 for WTs and Tgs, respectively, to account for an estimated attrition rate of approximately 15%. These group sizes (~20 or more) align well with behavioural analyses previously performed in this model[2], which were adequate to determine significant differences between genotypes in a cross-sectional analysis.

Group sizes for all modalities changed over the course of the study due to normal attrition with age, occasional malfunction of the camera and/or tracking software used for behavioural testing, and restricted access to our facility during COVID-19, particularly at the last time point. Additionally, if animals only had data from one time point for any modality (typically due to failure of quality control metrics at one time point combined with death prior to 18 months), that animal was removed from the dataset for that modality. This occurred for one rat within the MRS dataset, three rats within the MRI dataset, and two rats within the Barnes Maze dataset. Group sizes and average age at each timepoint, before and after exclusions during quality control, split by modality and sex, are summarized in **Supplementary Table 1.**

MRI methods

High-resolution 3D anatomical MR images were acquired using Rapid Acquisition with Relaxation Enhancement (RARE) using the following scan parameters: TR = 325 ms, echo spacing = 10.8 ms, RARE factor = 6, effective echo time = 32.4 ms, Field of View = 20.6 × 17.9 × 29.3 mm, matrix size = 256 × 180 × 157, slice thickness 17.9 mm (along the dorsal/ventral direction), readout along the rostral/caudal direction, scanner resolution = 114 µm isotropic, 19m35s acquisition time.

All pre-processing methodology is described in detail elsewhere. [3] Briefly, preprocessing was performed using minc-toolkit-v2 [4] with the MINC toolkit extras package (https://github.com/CoBrALab/minc-toolkit-extras), and the two-level model build Pydpiper module[5] was used to co-register the pre-processed images into a common space. First, an in-house rat MRI preprocessing script within the minc-toolkit-extras package developed by G.A.D. (https://github.com/CoBrALab/minc-toolkit-extras, rat-preprocessing-v4.sh) was used to perform the following sequential preprocessing steps: dimension reordering to standard MINC 2.0 ordering, image centring, whole image N4 bias field correction,[6,7] individual foreground mask generation using the Otsu method,[8] additional N4 bias field correction using the previously generated mask, registration to a Fischer 344 template average image, and a final N4 bias field correction using a template mask. After pre-processing, images were quality controlled by D.G. Images were visualised using the Display program in minc-toolkit-v2 and examined in each of the coronal, sagittal, and axial dimensions for motion artefacts, Gibbs ringing artefacts, and other image anomalies. 15 of a total 179 scans were excluded from further analysis. 7 rats (4Tg (1M/3F), 3 WT (2M/1F)) were excluded at 4-months, 1 at 10-months (1TgM), and 5 at 16-months (3Tg (1M/2F), 2WT (1M/1F)).

The remaining 164 scans were co-registered using the two-level model build pipeline in Pydpiper. This co-registration paradigm uses deformation-based morphometry techniques and is described fully by Friedel et al.[5] In brief, subject-specific starting averages are created by rigidly registering scans at different time points to a Fischer 344 atlas template,[9] followed by averaging. Iterative affine and non-linear registration and averaging is then repeated to produce an unbiased subject average. Subsequently, each subject specific average is rigidly aligned to the Fischer 344 atlas template space and the process is repeated to create an unbiased population average. This process creates deformation fields for each subject at each time point. The deformation fields can

then be used to estimate the Jacobian determinant at each voxel, which reflects the amount of expansion or compression required to deform each individual anatomical image to the subject average.[10] Deformation fields are then resampled into the common study space allowing comparison between subjects. This registration process generates two sets of Jacobian determinants, though only the relative Jacobian—composed solely of the non-linear mapping and thus reflects local or relative changes in voxel volume—was used for subsequent analysis. The Jacobian deformation fields were then blurred with a 400 micron full width half maximum Gaussian kernel to satisfy assumptions of normality required by the statistical models used to analyze the data.

Using the Fischer 344 rat atlas resampled into the common space of this study, the volumes of 120 regions were estimated using the *anatGetAll* function in RMINC_1.5.2.3.[11] This function computes the volume of a region by counting the number of voxels with a given label and multiplying the Jacobian with the voxel volume at each voxel.

MRS methods

Basis Set

The neurochemical basis set consisting of 18 simulated metabolite resonances and 9 macromolecule (MM) basis functions: alanine (Ala), aspartate (Asp), creatine (Cr), γ-aminobutyrate (GABA), glucose (Glc), glutamine (Gln), glutamate (Glu), glycerophosphocholine (GPC), glutathione (GSH), lactate (Lac), myo-Inositol (Ins), N-acetylaspartate (NAA), N-acetylaspartylglutamate (NAAG), phosphocholine (PCh), phosphocreatine (PCr), phosphoethanolamine (PE), serine (Ser), taurine (Tau), $MM_{0.89}$, $MM_{1.20}$, $MM_{1.39}$, $MM_{1.66}$, $MM_{2.02}$, $MM_{2.26}$, $MM_{2.97}$, $MM_{3.18}$, and $MM_{3.84}$. The subscript of each MM indicates the ppm value at which the peak appears in the MRS spectrum. We also report summed Cr+PCr (tCr), GPC+PCh (tCho), NAA+NAAG (tNAA) and Glu+Gln (Glx) and the ratios of Glu to Gln, Asp to Glu, and NAA to Ins.

Quality Control

To ensure high quality MRS data, we visually inspected each spectrum and removed three scans that generated the RFALSI 4 error during LCModel fitting, which is indicative of particularly

noisy data. These scans also had the lowest signal-to-noise ratio (SNR) of all 178 scans, with SNR of 13, 13, and 15, when the average was 23.13 (± 3.30, standard deviation).

The Cramer-Rao lower bound (CRLB) provided by LCModel was used as a measure of reliability of neurochemical quantification on a per-metabolite basis.[12] We employed a strict cut-off of 20% CRLB averaged across all scans, which resulted in the removal of GABA (CRLB 41.56), Serine (CRLB 33.59), and $MM_{3.18}$ (CRLB 44.96) from our analysis. Finally, after visual inspection of all graphed data, there were several extreme outliers. In two cases, the entire animal was removed from that time point, the first because MRS data was indicative of cancer (high total Choline, low NAA) and the second because the data points from that animal were outliers for all metabolites. A very conservative median absolute deviation (MAD) threshold of 3.5 [13,14] was applied to all remaining data points, resulting in the removal of 34 of 4671 data points, or 0.728% of the data.

Barnes Maze Testing

Due to the large number of animals in this study and the desire to identify early memory deficits in the TgF344-AD rat model, we followed a shortened and thus more cognitively challenging protocol while keeping the time per trial (3 minutes) and the maze set-up consistent with protocols designed for rats.[2,15] The maze consisted of a circular platform (122 cm diameter) constructed of PVC material with 20 holes (4 inches diameter) evenly spaced around the perimeter. A bottom layer was placed underneath such that 19 of 20 holes would have a false bottom (too shallow for the rat to enter but otherwise identical to the escape hole), and to allow for rotation of the the top of the maze between trials, ensuring rats could not track any scent cues or navigate using small markings on the maze surface itself. An escape box consisting of a dark chamber with a ramp was placed underneath one of the holes (target hole). The maze and escape box were coated with black waterproof epoxy paint to ensure they would be easily cleanable with Peroxyguard (hydrogen peroxide sanitizer). The maze was mounted on top of a large plastic box such that it stood 35" above the ground and was lit by two bright spotlights positioned to reduce shadows projected onto the maze. White curtains were drawn in a square around the maze and three simple visual cues were attached to three of the four curtain "walls".

In the week prior to Barnes Maze testing, rats were introduced to the escape box by placing them in it and allowing them to climb around and explore during their weekly handling session.

This exploration session was introduced after pilot testing where rats showed extreme hesitancy to enter the box during the training trials. Their fear of entering the escape hole was mitigated by introducing them to the box prior to testing and was therefore retained for the animals tested in this study.

The rats interacted with the Barnes Maze in three consecutive phases: habituation (2 trials over 1 day), training (5 trials over 2 days), and probe (1 trial). Before each phase, rats were acclimated to the testing room for one hour. For habituation trial 1, rats were placed directly into the escape cage underneath the maze and allowed to remain there or independently exit the escape cage and explore the maze for a maximum of 3 minutes. If the rat exited the box and re-entered it, or if 3 minutes elapsed without the rat exiting the box, the escape hole was immediately covered and the rat was kept in the box for 30 to 60 seconds to mimic the end of the trial before returning them to their home cage. For habituation trial 2, the process was similar but rats were placed directly in the centre of the maze and allowed to explore until they either entered the escape hole or 3 minutes had elapsed. Rats that did not enter the escape hole after 3 minutes were guided to the hole and nudged until they entered it or manually placed in the box if they did not enter it independently.

In the training phase, the escape hole location was changed by at least 90 degrees from its location during habituation. Rats were placed in the centre of the maze underneath an opaque bucket for 10 seconds so they would be facing a random direction upon the start of the trial. The trial began as soon as the experimenter lifted the bucket. Rats were given 3 minutes to locate and enter the escape hole. If they located the hole (end point of test) but turned away to explore further, or if they did not enter after 3 minutes, they were guided to the hole and nudged to enter, or manually placed in the hole if they did not enter independently. The hole was covered and the rat remained in the escape hole for 30-60 seconds before being returned to their home cage. The maze and escape box was cleaned in between each rat, and the top of the maze was rotated between cages of rats. This process typically took 5 minutes per rat and was performed with 4-6 rats at a time, providing a 20-30 minute inter-trial interval. Training was repeated 3 times on day 1, and 2 times the subsequent day for a total of 5 training trials.

The probe trial, used to assess long-term spatial reference memory (Pitts, 2018), took place 48 hours after the last training trial. The escape cage was replaced with a false bottom such that all 20 holes appeared the same and escape was not possible. Rats were again placed in the centre of

the maze under an opaque bucket and, upon removal of the bucket, were allowed to explore the maze for 3 minutes before being returned to their home cage. The maze was cleaned and the maze top was rotated between each rat.

Statistical Analysis

Base Packages in R

The following base packages were attached in R: splines, stats, graphics, grDevices, utils, datasets, methods, base; other attached packages: effects_4.4-4,[16] RColorBrewer_1.1-2,[17] readxl_1.3.1,[18] lmerTest_3.1-0,[19] lme4_1.1-23,[20] tidyverse_1.3.0,[21] RMINC_1.5.2.3[11]).

Linear model comparison

Akaike information criterion (AIC) comparisons were performed for both volume and metabolite data to determine if age was best modelled using a linear or quadratic age term, given that non-linear change with age has previously been demonstrated with brain volumes[22–24] and metabolite concentrations.[25] AIC comparison using a threshold of Δ_i (AIC-AIC_{min})>4 [26] demonstrated that brain volumes were best fit using a quadratic age term (poly(age,2)), while all metabolite concentrations were best fit using a linear age term.

Final linear models

The full linear models for longitudinal analysis of brain structure and chemistry are shown below. For MRS data only (models 2 and 4), the fixed effect of water linewidth (water.lw) was included to control for the effect of linewidth on metabolite concentration estimates.[27] A weighting factor of the inverse absolute CRLB for each metabolite (1/metabolite.sdab) accounted for differences in fitting reliability between samples and allowed us to include all observations with CRLB <999.

Model 1: lmer(volume) ~ poly(age,2)*genotype +sex + (1|subject)

Model 2: lmer(metabolite) ~ age*genotype+sex +water.lw+(1|subject), weights=1/metabolite.sdab

Model 3: lmer(volume) ~ poly(age,2)*genotype*sex + (1|subject)

Model 4: lmer(metabolite) ~ age*genotype*sex +water.lw+(1|subject), weights=1/metabolite.sdab

Supplemental Material Titles and Legends

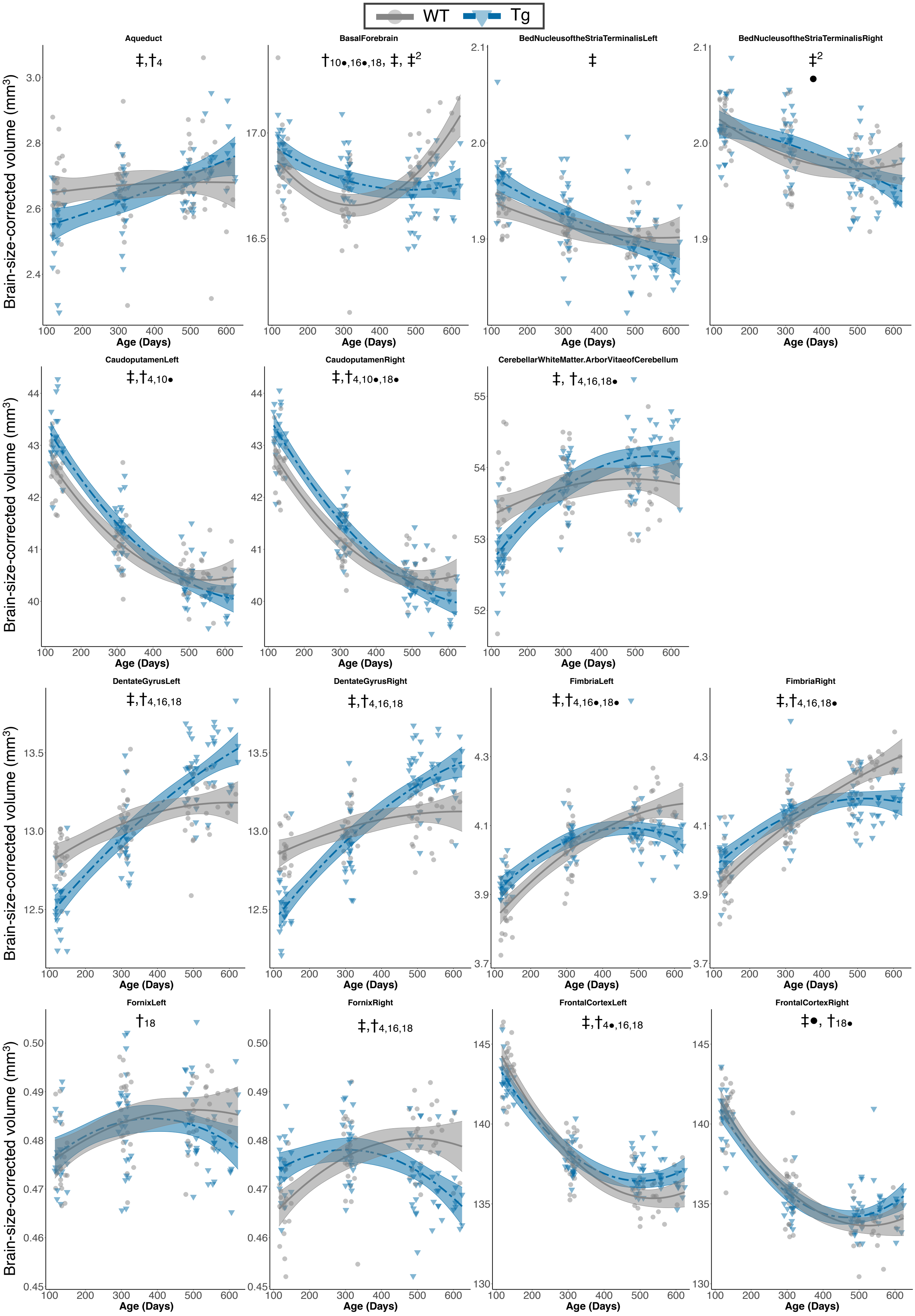
WT
Tg
Aqueduct
‡,†4
BasalForebrain
†10●,16●,18, ‡, ‡2
BedNucleusoftheStriaTerminalisLeft
‡
BedNucleusoftheStriaTerminalisRight
‡2
●
CaudoputamenLeft
‡,†4,10●
CaudoputamenRight
‡,†4,10●,18●
CerebellarWhiteMatter.ArborVitaeofCerebellum
‡, †4,16,18●
DentateGyrusLeft
‡,†4,16,18
DentateGyrusRight
‡,†4,16,18
FimbriaLeft
‡,†4,16●,18●
FimbriaRight
‡,†4,16,18●
FornixLeft
†18
FornixRight
‡,†4,16,18
FrontalCortexLeft
‡,†4●,16,18
FrontalCortexRight
‡●, †18●
Brain-size-corrected volume (mm3)
Age (Days)

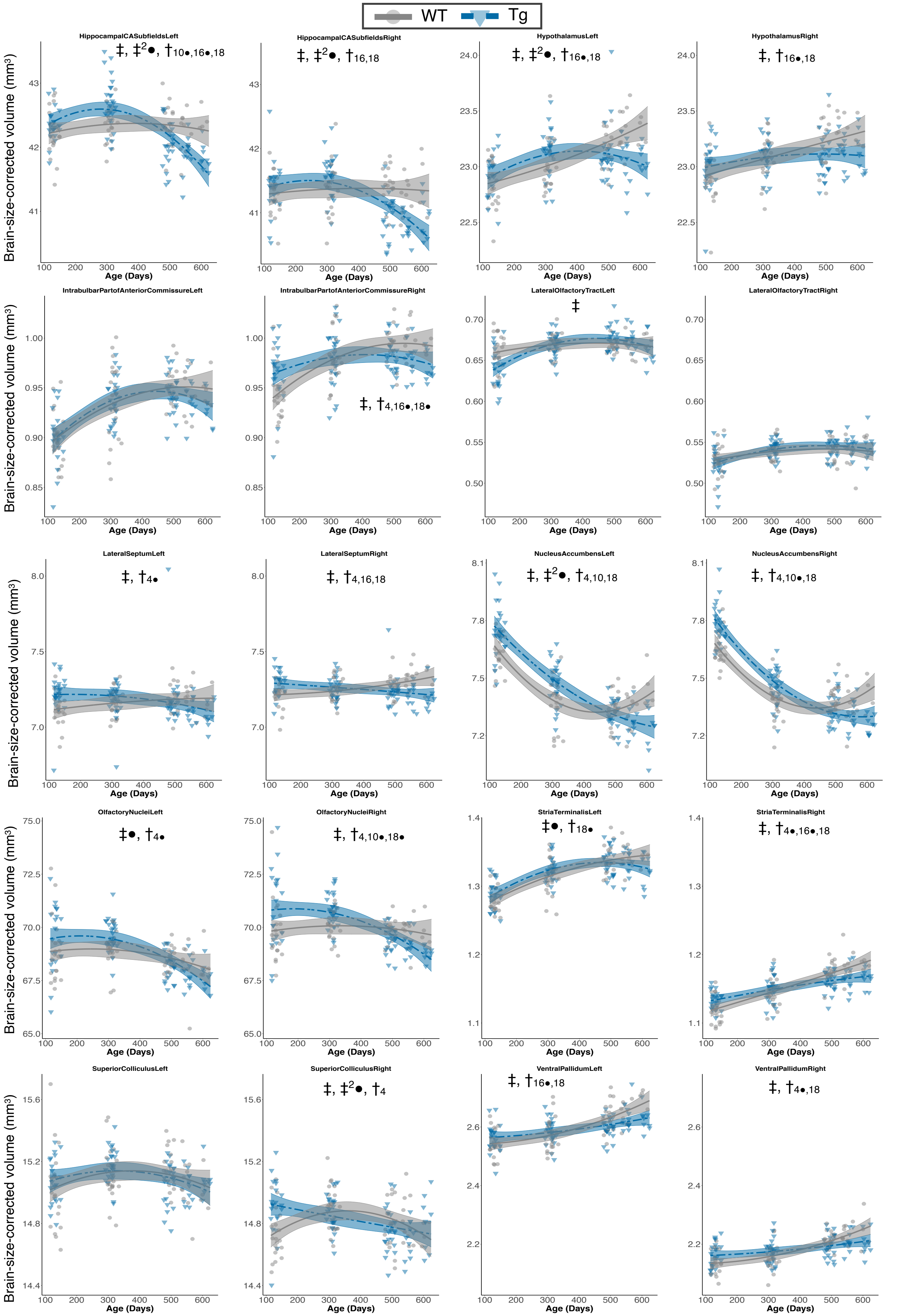

WT
Tg
Brain-size-corrected volume (mm³)
Age (Days)
HippocampalCASubfieldsLeft
‡, ‡2●, †10●,16●,18
HippocampalCASubfieldsRight
‡, ‡2●, †16,18
HypothalamusLeft
‡, ‡2●, †16●,18
HypothalamusRight
‡, †16●,18
IntrabulbarPartofAnteriorCommissureLeft
IntrabulbarPartofAnteriorCommissureRight
‡, †4,16●,18●
LateralOlfactoryTractLeft
‡
LateralOlfactoryTractRight
LateralSeptumLeft
‡, †4●
LateralSeptumRight
‡, †4,16,18
NucleusAccumbensLeft
‡, ‡2●, †4,10,18
NucleusAccumbensRight
‡, †4,10●,18
OlfactoryNucleiLeft
‡●, †4●
OlfactoryNucleiRight
‡, †4,10●,18●
StriaTerminalisLeft
‡●, †18●
StriaTerminalisRight
‡, †4●,16●,18
SuperiorColliculusLeft
SuperiorColliculusRight
‡, ‡2●, †4
VentralPallidumLeft
‡, †16●,18
VentralPallidumRight
‡, †4●,18

Supplementary Figures 1 and 2: Visualization of the longitudinal volume trajectories of all brain structures that display a significant age by genotype interaction. Brain-size-corrected volumes were predicted via linear mixed effects modelling using a second order age by genotype interaction with sex covaried and a random intercept for each subject. Multiple comparisons were corrected for using a 5% false discovery rate. The mixed effects model used to fit the data is represented by a line of best fit and 95% interval (shaded). Each data point represents a single rat. Data corresponding to wildtype (WT) rats are shown using grey circles and a solid line of best fit, while data corresponding to TgF344-AD (Tg) rats are shown using blue triangles and a dashed line of best fit. Significance symbols are shown for the linear age by genotype interaction term (‡), quadratic age by genotype interaction term ($‡^2$), and the main effect of genotype (†) at each time point as determined by the age-centered models, with the subscript denoting at which age the genotype effect was significant. ● denotes an effect significant at the original p-value level but not after FDR correction.

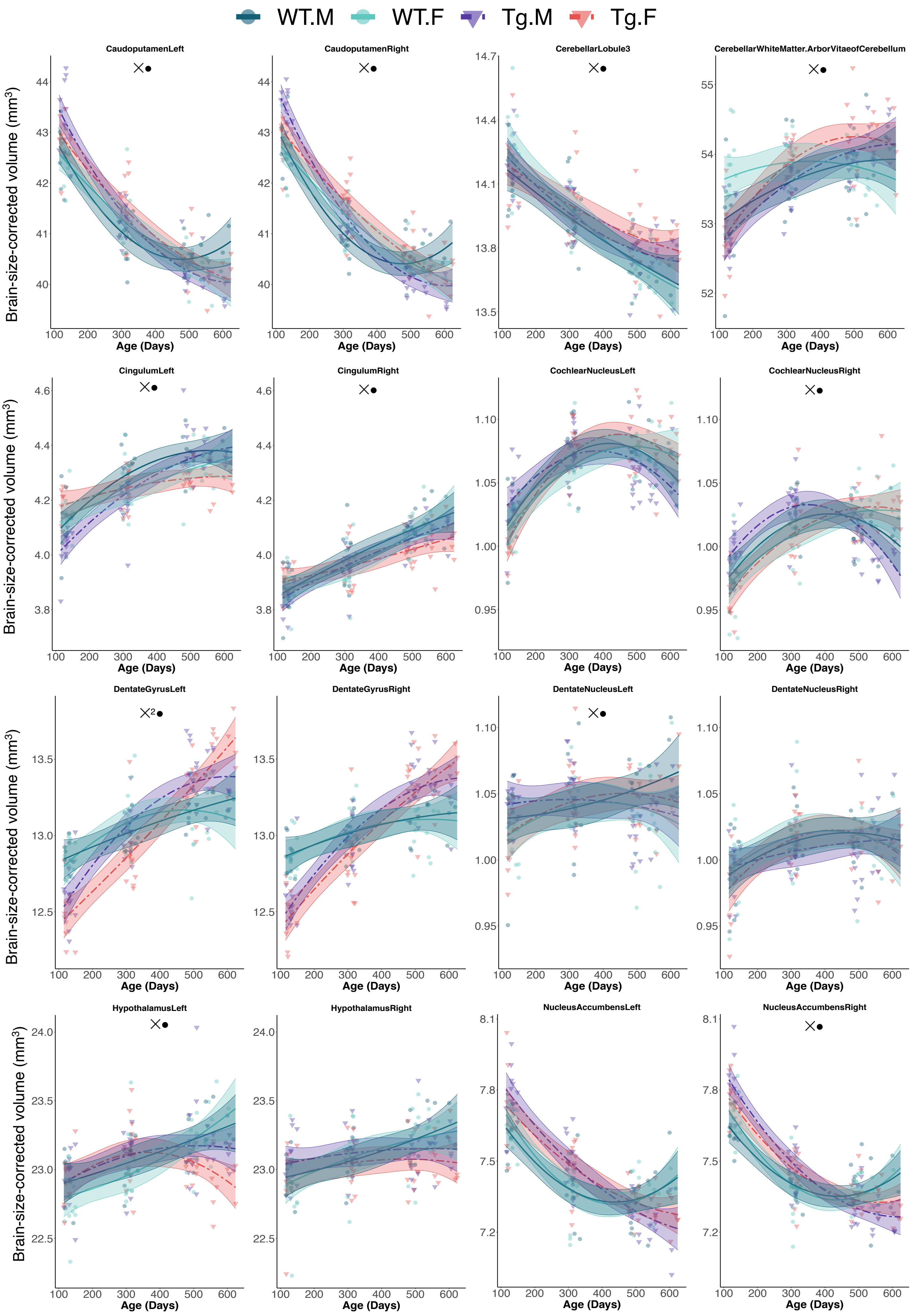
WT.M
WT.F
Tg.M
Tg.F
CaudoputamenLeft
CaudoputamenRight
CerebellarLobule3
CerebellarWhiteMatter.ArborVitaeofCerebellum
CingulumLeft
CingulumRight
CochlearNucleusLeft
CochlearNucleusRight
DentateGyrusLeft
DentateGyrusRight
DentateNucleusLeft
DentateNucleusRight
HypothalamusLeft
HypothalamusRight
NucleusAccumbensLeft
NucleusAccumbensRight
Brain-size-corrected volume (mm³)
Age (Days)
100 200 300 400 500 600

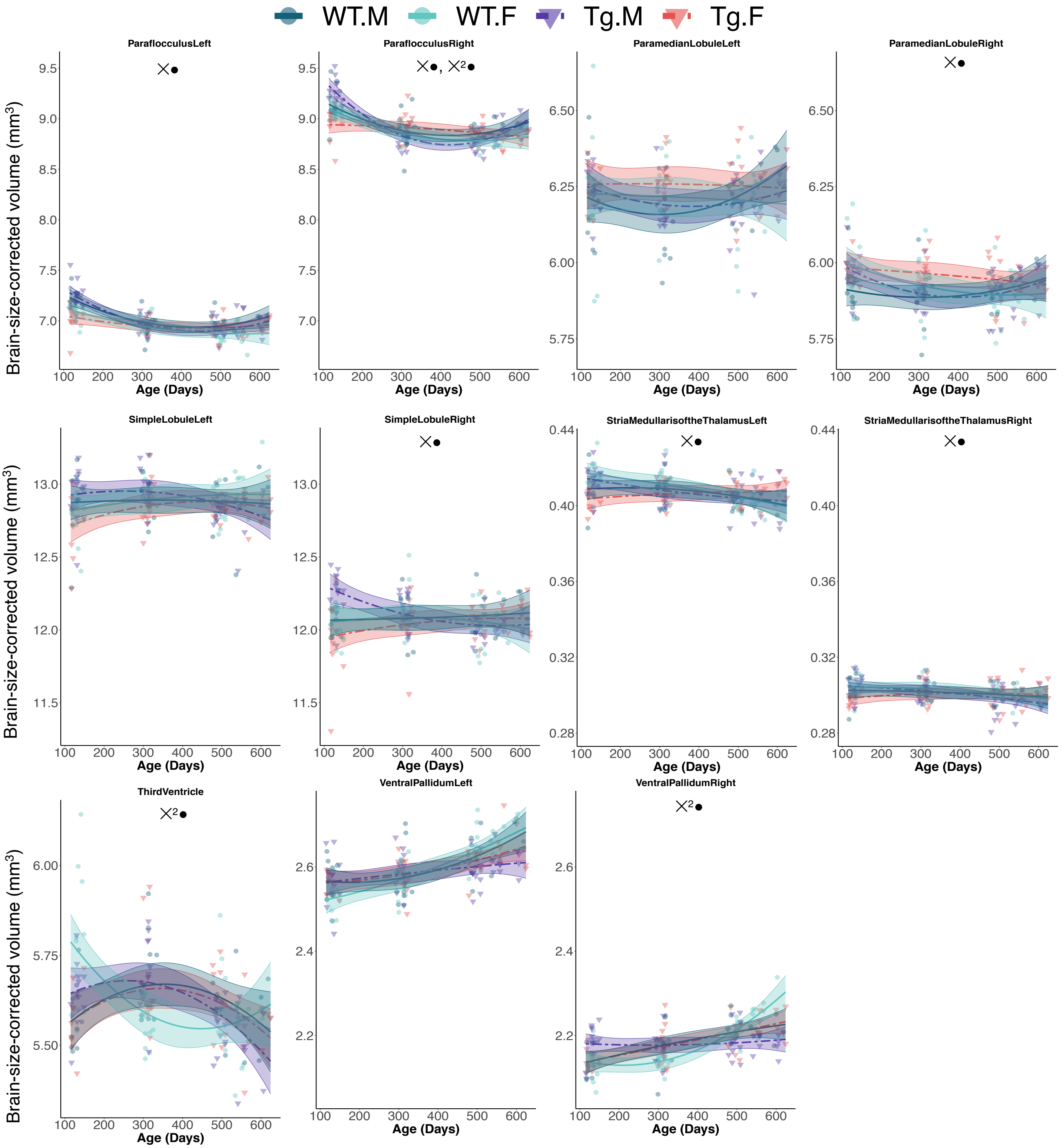

WT.M
WT.F
Tg.M
Tg.F
ParaflocculusLeft
ParaflocculusRight
ParamedianLobuleLeft
ParamedianLobuleRight
SimpleLobuleLeft
SimpleLobuleRight
StriaMedullarisoftheThalamusLeft
StriaMedullarisoftheThalamusRight
ThirdVentricle
VentralPallidumLeft
VentralPallidumRight
Brain-size-corrected volume (mm³)
Age (Days)

Supplementary Figures 3 and 4: Visualization of the longitudinal volume trajectories of all brain structures that display a significant age by genotype by sex interaction, prior to FDR correction. Brain-size-corrected volumes were predicted via linear mixed effects modelling using a second order age by genotype by sex interaction and a random intercept for each subject. Multiple comparisons were corrected for using a 5% false discovery rate. The mixed effects model used to fit the data is represented by a line of best fit and 95% interval (shaded). Each data point represents a single rat. Data corresponding to wildtype (WT) rats are shown using circles and a solid line of best fit, while data corresponding to TgF344-AD (Tg) rats are shown using triangles and a dashed line of best fit. Significance symbols are shown for the linear age by genotype by sex interaction term (X) and quadratic age by genotype interaction term (X^2). A single symbol indicates an adjusted p-value <0.05. ● denotes an effect significant at the original p-value level but not after FDR correction. Abbreviations: wildtype male, WT.M; wildtype female, WT.F; TgF344-AD male, Tg.M; TgF344-AD female, Tg.F.

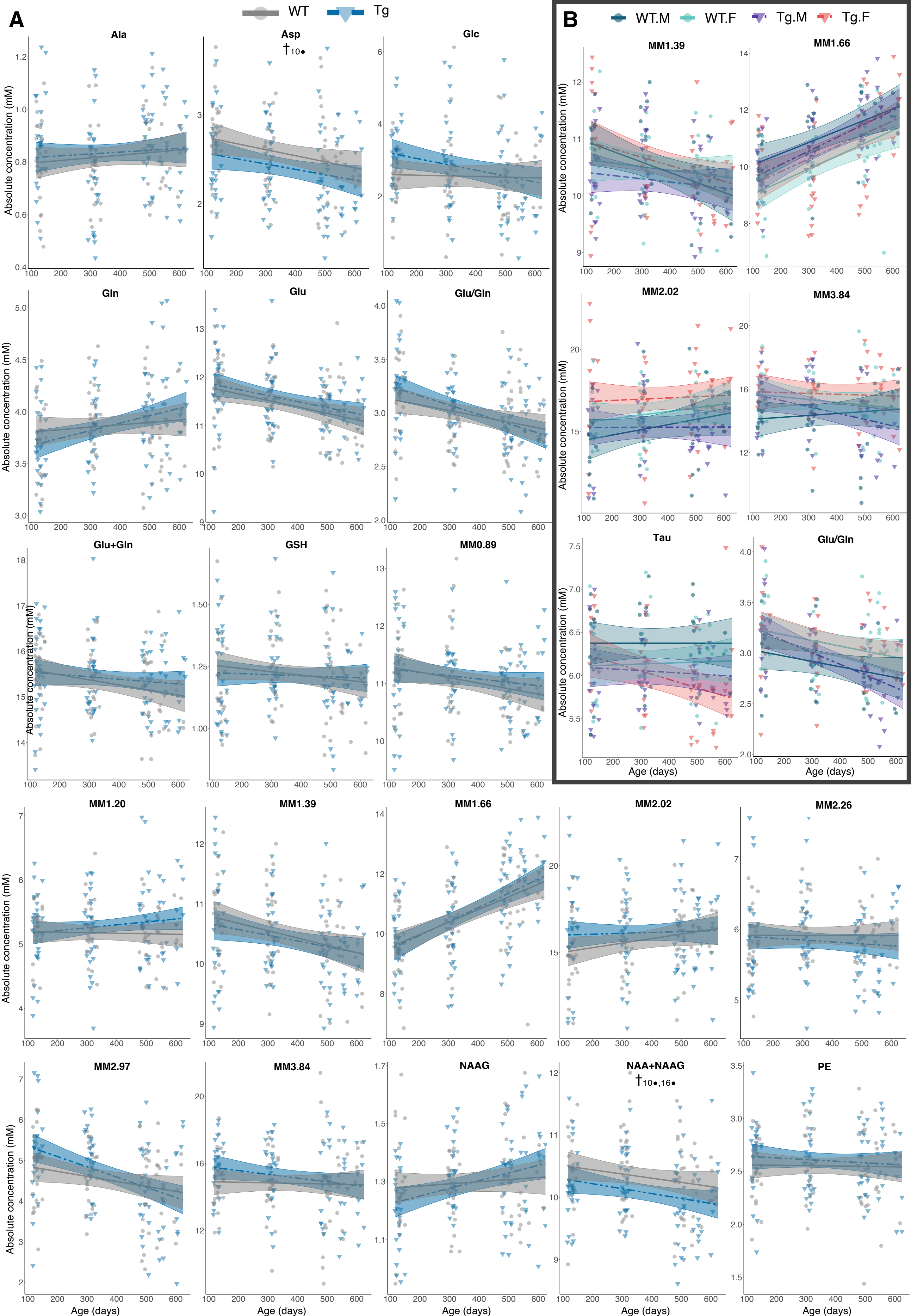
A
WT
Tg
Ala
Asp
†10•
Glc
Gln
Glu
Glu/Gln
Glu+Gln
GSH
MM0.89
MM1.20
MM1.39
MM1.66
MM2.02
MM2.26
MM2.97
MM3.84
NAAG
NAA+NAAG
†10•,16•
PE
B
WT.M
WT.F
Tg.M
Tg.F
MM1.39
MM1.66
MM2.02
MM3.84
Tau
Glu/Gln
Absolute concentration (mM)
Age (days)

Supplementary Figure 5: Longitudinal trajectory of neurochemicals in TgF344-AD and wildtype rats, with some split by sex. Absolute concentration (mM) of neurochemicals were predicted via linear mixed effects modelling using either **A)** an age by genotype interaction with sex covaried (model 3), or **B)** a three-way interaction between age, genotype, and sex (model 4). In both cases, water linewidth was also covaried and a random intercept for each subject was included. Multiple comparisons were corrected for using a 5% false discovery rate. The mixed effects model used to fit the data is represented by a line of best fit and 95% interval (shaded). Each data point represents a single rat. Data corresponding to WT rats are shown using circles and a solid line of best fit, while data corresponding to TgF344-AD rats are shown using triangles and a dashed line of best fit, respectively. Significance symbols in **A** are shown for the linear age by genotype interaction term (‡) and the main effect of genotype (†) at each time point as determined by the age-centered models, with the subscript denoting at which age the genotype effect was significant. The significance symbol for the age by genotype by sex interaction term (X) from model 4 shown in **B** was not necessary as none of the results were significant. ● denotes an effect significant at the p-value level but not after FDR correction. Abbreviations: Alanine (Ala), Aspartate (Asp), Glucose (Glc), Glutamine (Gln), Glutamate (Glu), Glutathione (GSH), N-acetylaspartate (NAA), N-acetylaspartylglutamate (NAAG), Phosphoethanolamine (PE), Taurine (Tau), Macromolecule (MM). The subscript of each MM indicates the ppm value at which the peak appears in the MRS spectrum.

Supplementary Table 1. Summary of subject demographic data at each timepoint, split by modality and sex. Number of subjects is shown both before and after exclusions during quality control. Squares highlighted in pink indicate where quality control reduced the number of rats with usable data. Average age in days is shown, with the standard deviation included after the plus minus symbol. Abbreviations: M, male; F, female.

Supplementary Table 2: LME_MRI: Akaike's information criterion (AIC) analysis and linear model summary for 120 brain volumes. AIC was used to determine whether or not the majority of structures were better fit using a linear versus quadratic age term. Model A predicted volume via poly(age,1)*genotype + sex + (1|subject) while model B predicted volume via poly(age,2)*genotype*sex + (1|subject). For each structure, delta AIC (AIC_i-$AIC_{minimum}$) > 4 was indicative of a better fit using a quadratic age term, which was the case for the majority of structures. Model B was therefore applied to all brain volumes and each term from model B is shown under the primary hypothesis heading, whereby the model terms of interest were the linear (poly(age,2)1:genotypeTg)) and quadratic (poly(age,2)2:genotypeTg)) interaction terms. Model B was also run in the form of four age-centered models, wherein the model term of interest was the main effect of genotype, denoted by genotypeTg(age). Standard beta values, standard error, and p-values are shown for all model terms, while model terms of interest are highlighted with a black border and include a column with adjusted p-values after 5% FDR correction. For these terms of interest, p-values and adjusted p-values that meet the significance threshold of 0.05 are highlighted in blue, while those between 0.05 and 0.1 are highlighted in grey. A secondary analysis was also performed whereby the model included a 3-way interaction between quadratic age, genotype, and sex, with a random intercept per subject. The model terms of interest for this analysis were genotypeTg:sexF, poly(age,2)1:genotypeTg:sexF, and poly(age,2)2:genotypeTg:sexF. All model terms are shown under the secondary hypothesis heading.

Supplementary Table 3: MRI_volume_summary_mm3: Summary of brain volumes in mm^3 measured longitudinally in TgF344-AD (Tg) rats and wildtype (WT) controls, as well as split by sex. Volumes are expressed as mean ± standard deviation. N indicates the number of subjects per group. Abbreviations: WT male (WTM), WT female (WTF), Tg male (TgM), Tg female (TgF).

Supplementary Table 4: LME_MRS: CRLB values, Akaike's information criterion (AIC) analysis, and linear model summary for 27 neurochemicals. Cramer-Rao Lower Bounds were averaged across all scans and used as a quality control metric with a strict threshold of 20%, resulting in the removal of GABA (CRLB 41.56), Serine (CRLB 33.59), and $MM_{3.18}$ (CRLB 44.96) from further analysis. AIC was used to determine whether or not the majority of neurochemicals were better fit using a linear versus quadratic age term. Model A predicted concentration via poly(age,1)*genotype + sex + water.linewidth (lw) + (1|subject) while model 2 predicted concentration via poly(age,2)*genotype*sex + water.lw + (1|subject). For each neurochemical, delta AIC (AIC_i-$AIC_{minimum}$) > 4 was indicative of a better fit using a quadratic age term. All neurochemicals demonstrated a better fit using Model A with a linear age term. Model A was therefore applied to all neurochemicals and each model term is shown under the primary hypothesis heading, whereby the model term of interest was the age by genotype interaction term (age:genotypeTg). Model A was also applied in the form of four age-centered models, whereby the term of interest was the main effect of genotype, evaluated at four time points (genotypeTg(age)). Standard beta values, standard error, and p-values are shown for all model terms, while model terms of interest are highlighted with a black border and include a column with adjusted p-values after 5% FDR correction. For these terms of interest, p-values and adjusted p-values that meet the significance threshold of 0.05 are highlighted in blue, while those between 0.05 and 0.1 are highlighted in grey. A secondary analysis was also performed whereby the model included a 3-way interaction between age, genotype, and sex, with water linewidth covaried and a random intercept per subject. The model terms of interest for this analysis were genotypeTg:sexF and age:genotypeTg:sexF. All model terms are shown under the secondary hypothesis heading.

Supplementary Table 5: MRS_conc_summary_mMol: Summary of neurochemical concentrations in mMol measured longitudinally in TgF344-AD (Tg) rats and wildtype (WT) controls, as well as split by sex. Concentrations are expressed as mean ± standard deviation. 'n' indicates the number of subjects per group. Abbreviations: WT male (WTM), WT female (WTF), Tg male (TgM), Tg female (TgF).

Supplementary Table 6: Barnes_stats: All statistics run on Barnes Maze probe data for both primary (main effect of genotype) and secondary analyses (genotype by sex). The statistical test for each of the five metrics (% time in target quadrant, % time in target holes, % success, number

of holes searched, and speed) is shown, along with the effect size (either cohen's d or standardized beta) for all terms in the model. Statistics are split by time point. Bonferroni correction was applied at each time point for primary analyses and secondary analysis separately. For primary analyses, the number of tests run per time point was 7 (4 one-sample t-tests and 3 linear models) so the significance threshold was 0.05/7=0.00714. For secondary analyses the number of tests per time point was 11 (8 one-sample t-tests and 3 linear models), so the significance threshold was 0.05/11 = 0.00455. P-values that reached the appropriate significance threshold are highlighted in blue.

Supplementary Table 7: Barnes_data_summary: Summary of Barnes maze statistics measured at 4 time points in TgF344-AD (Tg) rats and wildtype (WT) controls, as well as split by sex. N represents the number of subjects per group. For each metric the average, standard deviation (SD), standard error (SE), and 95% confidence interval (CI) are shown. Abbreviations: WT male (WTM), WT female (WTF), Tg male (TgM), Tg female (TgF).

References

1. Lerch JP, Gazdzinski L, Germann J, Sled JG, Henkelman RM, Nieman BJ. Wanted dead or alive? The tradeoff between in-vivo versus ex-vivo MR brain imaging in the mouse. *Front Neuroinform*. 2012;6:6.
2. Cohen RM, Rezai-Zadeh K, Weitz TM, et al. A transgenic Alzheimer rat with plaques, tau pathology, behavioral impairment, oligomeric aβ, and frank neuronal loss. *J Neurosci*. 2013;33(15):6245-6256.
3. Fowler C, Goerzen D, Madularu D, Devenyi GA, Mallar Chakravarty M, Near J. Longitudinal characterization of neuroanatomical changes in the Fischer 344 rat brain during normal aging and between sexes. *bioRxiv*. Published online April 13, 2021:2021.04.12.439510. doi:10.1101/2021.04.12.439510
4. Vincent RD, Neelin P, Khalili-Mahani N, et al. MINC 2.0: A Flexible Format for Multi-Modal Images. *Front Neuroinform*. 2016;10:35.
5. Friedel M, van Eede MC, Pipitone J, Chakravarty MM, Lerch JP. Pydpiper: a flexible toolkit for constructing novel registration pipelines. *Front Neuroinform*. 2014;8:67.
6. Sled JG, Pike GB. Standing-wave and RF penetration artifacts caused by elliptic geometry: an electrodynamic analysis of MRI. *IEEE Trans Med Imaging*. 1998;17(4):653-662.
7. Tustison NJ, Avants BB, Cook PA, et al. N4ITK: improved N3 bias correction. *IEEE Trans Med Imaging*. 2010;29(6):1310-1320.
8. Otsu N. A Threshold Selection Method from Gray-Level Histograms. *IEEE Trans Syst Man Cybern*. 1979;9(1):62-66.
9. Goerzen D, Fowler C, Devenyi GA, et al. An MRI-Derived Neuroanatomical Atlas of the Fischer

344 Rat Brain. *Sci Rep*. 2020;10(1):6952.

10. Chung MK, Worsley KJ, Paus T, et al. A unified statistical approach to deformation-based morphometry. *Neuroimage*. 2001;14(3):595-606.

11. Lerch J, Hammill C, van Eede M, Cassel D. Statistical Tools for Medical Imaging NetCDF (MINC) Files. R package version 1.5.2.3. Published 2017. http://mouse-imaging-centre.github.io/RMINC/

12. Kreis R. The trouble with quality filtering based on relative Cramér-Rao lower bounds. *Magn Reson Med*. 2016;75(1):15-18.

13. Iglewicz B, Hoaglin DC. *How to Detect and Handle Outliers*. Vol 16. (Mykytka EF, Westergard S, Wall A, eds.). ASQC/Quality Press; 1997.

14. Leys C, Ley C, Klein O, Bernard P, Licata L. Detecting outliers: Do not use standard deviation around the mean, use absolute deviation around the median. *J Exp Soc Psychol*. 2013;49(4):764-766.

15. Pitts MW. Barnes Maze Procedure for Spatial Learning and Memory in Mice. *Bio Protoc*. 2018;8(5). doi:10.21769/bioprotoc.2744

16. Fox J, Weisberg S. An R Companion to Applied Regression. Published online 2019. https://socialsciences.mcmaster.ca/jfox/Books/Companion/index.html

17. Neuwirth E. *RColorBrewer: ColorBrewer Palettes*.; 2014. https://CRAN.R-project.org/package=RColorBrewer

18. Wickham H, Bryan J. *Readxl: Read Excel Files*.; 2019. https://CRAN.R-project.org/package=readxl

19. Kuznetsova A, Brockhoff PB, Christensen RHB. lmerTest Package: Tests in Linear Mixed Effects Models. *Journal of Statistical Software, Articles*. 2017;82(13):1-26.

20. Bates D, Mächler M, Bolker B, Walker S. Fitting Linear Mixed-Effects Models Using lme4. *Journal of Statistical Software, Articles*. 2015;67(1):1-48.

21. Wickham H, Averick M, Bryan J, et al. Welcome to the tidyverse. *J Open Source Softw*. 2019;4(43):1686.

22. Pfefferbaum A, Rohlfing T, Rosenbloom MJ, Chu W, Colrain IM, Sullivan EV. Variation in longitudinal trajectories of regional brain volumes of healthy men and women (ages 10 to 85 years) measured with atlas-based parcellation of MRI. *Neuroimage*. 2013;65:176-193.

23. Kong V, Devenyi GA, Gallino D, et al. Early-in-life neuroanatomical and behavioural trajectories in a triple transgenic model of Alzheimer's disease. *Brain Struct Funct*. 2018;223(7):3365-3382.

24. Tullo S, Patel R, Devenyi GA, et al. MR-based age-related effects on the striatum, globus pallidus, and thalamus in healthy individuals across the adult lifespan. *Hum Brain Mapp*. 2019;40(18):5269-5288.

25. Fowler CF, Madularu D, Dehghani M, Devenyi GA, Near J. Longitudinal quantification of metabolites and macromolecules reveals age- and sex-related changes in the healthy Fischer 344 rat brain. *Neurobiol Aging*. 2020;101:109-122.

26. Burnham KP, Anderson DR. Multimodel Inference: Understanding AIC and BIC in Model Selection. *Sociol Methods Res*. 2004;33(2):261-304.

27. Bartha R. Effect of signal-to-noise ratio and spectral linewidth on metabolite quantification at 4 T. *NMR Biomed*. 2007;20(5):512-521.
